# Repetitive Two-Step Method for *o*,*o*,*p*- and *o*,*p*-Oligophenylene Synthesis through Pd-Catalyzed Cross-Coupling of Hydroxyterphenylboronic Acid

**DOI:** 10.3390/molecules181215207

**Published:** 2013-12-10

**Authors:** Miyuki Yamaguchi, Takeshi Kimura, Naomi Shinohara, Kei Manabe

**Affiliations:** School of Pharmaceutical Sciences, University of Shizuoka, 52-1 Yada, Suruga-ku, Shizuoka 422-8526, Japan; E-Mails: yamaguchim@u-shizuoka-ken.ac.jp (M.Y.); t.kimura@ki-chem.co.jp (T.K.); m08060@u-shizuoka-ken.ac.jp (N.S.)

**Keywords:** palladium, Suzuki-Miyaura coupling, oligoarene, nonaflate

## Abstract

A repetitive two-step method involving the Pd-catalyzed Suzuki-Miyaura coupling of hydroxyterphenylboronic acid and the subsequent nonaflation of the hydroxy group has been developed for the synthesis of oligophenylenes. This method readily afforded *o*,*o*,*p*- and *o*,*p*-oligophenylenes with defined chain lengths. X-ray crystallography was employed to obtain the structure of the *o*,*p*-oligophenylene 9-mer.

## 1. Introduction

Oligophenylenes, which are composed of benzene rings connected through single bonds, have attracted considerable attention as an important class of oligomers [1,2,3,4,5]. Oligophenylenes are widely used architectures in electronic devices [6] and are employed as self-assembling [7,8,9], biologically active [10], and catalytic molecules [11,12]. In addition, oligophenylenes can be used as building blocks for the synthesis of well-defined graphite subunits [13]. The conformational and electronic properties of oligophenylenes have also been subjects of intensive research [14,15,16,17,18]. Owing to such widespread interest, it is crucial to develope synthetic methods that can produce oligophenylenes with the desired chain length and connectivity pattern. Although one-step syntheses of long polyphenylenes have been reported [2], the resulting compounds are obtained as mixtures of varying chain lengths rather than a single species. In order to synthesize oligophenylenes with structural homogeneity, stepwise synthetic methods are necessary. However, while several synthetic protocols for the preparation of such molecules have been reported [11,19], the development of efficient methods still remains a challenging task. 

**Scheme 1:**
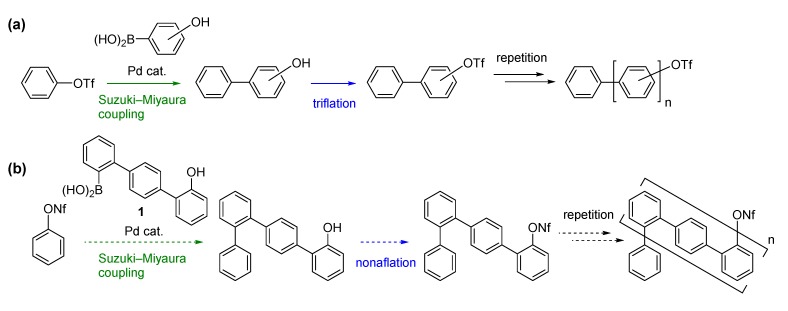
Repetitive two-step method for oligophenylene synthesis. (**a**) Our previous method; (**b**) Method based on the use of hydroxyterphenylboronic acid. Tf = –SO_2_CF_3_, Nf = –SO_2_C_4_F_9_.

We previously developed a method for oligophenylene synthesis via repetitive Suzuki-Miyaura coupling [20,21] of hydroxyphenylboronic acids with sebsequent triflation of the hydroxy group (Scheme 1a) [22,23]. While this repetitive two-step method realizes the facile synthesis of a variety of oligophenylenes with a specific chain length and different functional groups, it was only able to introduce one benzene unit in a single Suzuki-Miyaura coupling step. Based on this two-step protocol, Hartley *et al.* developed an improved method in which two benzene units could be introduced in a single step [15]. However, the development of more efficient methods for synthesizing longer oligophenylenes is still needed. Herein, we describe a new version of the repetitive two-step method, which utilizes hydroxyterphenylboronic acid **1** and enables the introduction of *three* benzene units in one step (Scheme 1b). By employing this method, oligophenylenes with two different types of connectivity pattern, *o*,*o*,*p*- and *o*,*p*-oligophenylenes, were successfully synthesized in a small number of steps.

## 2. Results and Discussion

**Scheme 2:**
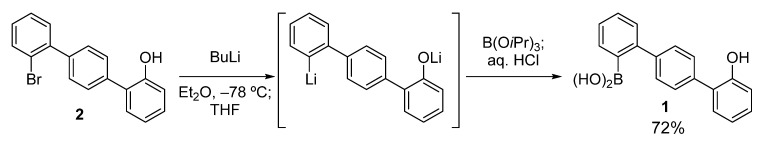
Preparation of hydroxyterphenylboronic acid **1**.

The key boronic acid, hydroxyterphenylboronic acid **1**, was easily prepared according to the route shown in Scheme 2. Compound **2** [24] was treated with BuLi in Et_2_O at −78 °C, and then THF was added. This sequential use of the two solvents (Et_2_O and THF) ensured satisfactory conversion to the dilithiated compound, without the formation of a significant amount of the byproduct protonated at the lithiated carbon [25]. This was possible because the Li–Br exchange occurred only after the addition of THF [26]. The dilithiated compound was then boronated to give **1** in good yield (72%).

Boronic acid **1** was first applied to the synthesis of *o*,*o*,*p*-oligophenylenes, composed of benzene rings connected in the order of *ortho*, *ortho*, and then *para*. These were envisioned to make up a new structural motif of folding oligophenylenes [27]. While *o*,*o*,*p*-oligophenylenes could be synthesized using our previous reported method [27] involving the C–H arylation of bipheny-2-ols as the key step, the present method using **1** would be more efficient for synthesis of longer oligomers. 

**Scheme 3:**
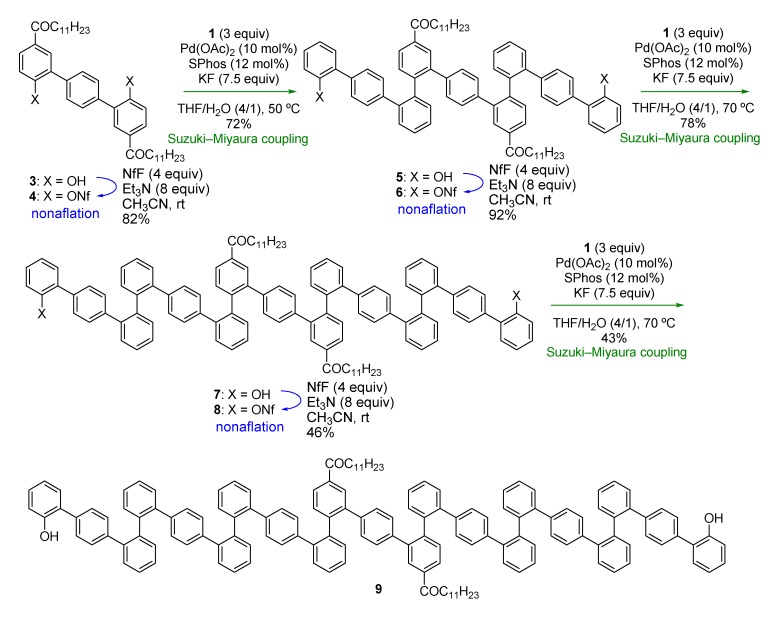
Synthesis of *o*,*o*,*p*-oligophenylenes.

Thus, we started with compound **3** (Scheme 3), with dodecanoyl groups introduced in order to increase the solubility of the oligomers in organic solvents. While the triflyl group (Tf, –SO_2_CF_3_) was used to activate hydroxy groups in the previous work (Scheme 1a), we decided to use the nonaflyl group (Nf, –SO_2_C_4_F_9_) [27,28], as this is more stable against O–SO_2_ bond cleavage [29,30] and can be prepared with NfF, which is usually less expensive than the commonly used triflating agent, Tf_2_O. 

Nonaflation of **3** gave bisnonaflate **4**, which was then subjected to Suzuki-Miyaura coupling with **1** in the presence of a Pd/SPhos catalyst [31]. Double Suzuki-Miyaura coupling introduced two terphenyl units to give **5** in good yield (72%). Repetition of the nonaflation/Suzuki-Miyaura-coupling sequence twice afforded symmetric *o*,*o*,*p*-oligophenylene **9**. It should be emphasized that only six steps were required to synthesize 21-mer oligophenylene **9** from **3**. Although these *o*,*o*,*p*-oligophenylenes showed complicated ^1^H- and ^13^C-NMR spectra because of the existence of rotamers, even at 100 °C, high-resolution mass spectrometry (HRMS) and high performance liquid chromatography (HPLC) analysis verified the identities and the purities of the oligomers. 

We next turned our attention to combining the previous and present strategies (Scheme 1a,b) to facilitate synthesis of another type of oligophenylenes. *o,p*-Oligophenylenes, which are used as precursors in bottom-up synthesis of graphene nanoribbons [32], were selected as the model target [33] in order to demonstrate the feasibility of the combined strategy. 

**Scheme 4:**
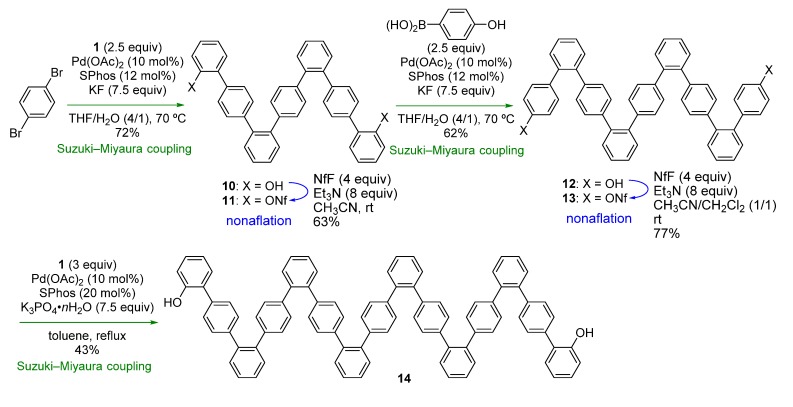
Synthesis of *o*,*p*-oligophenylenes.

The synthesis commenced using 1,4-dibromobenzene, which was subjected to Suzuki-Miyaura coupling with **1** (Scheme 4). Although the reaction was slow at rt, raising the temperature to 70 °C resulted in a good yield of 7-mer **10** (72%). After nonaflation, Suzuki-Miyaura-coupling with 4-hydroxyphenylboronic acid was conducted, affording 9-mer **12**. While nonaflation of **12** in CH_3_CN resulted in a low yield due to the low solubility of **12** in this particular solvent, use of a mixed solvent (CH_3_CN/CH_2_Cl_2_) improved the yield to 77%. For the final Suzuki-Miyaura-coupling step, it was necessary to change the reaction conditions, as the low solubility of **13** in THF/H_2_O hampered the reaction under the previous employed conditions. Finally, we found that the use of K_3_PO_4_∙nH_2_O in toluene gave 15-mer **14** in a modest yield (43%). In contrast to the *o*,*o*,*p*-oligophenylenes, rotamers were not observed in the NMR spectra of the *o*,*p*-oligophenylenes at room temperature. The synthesis shown in Scheme 4 clearly demonstrates that *o*,*p*-oligophenylenes with a specific chain length can be easily synthesized through this strategy.

**Figure 1:**
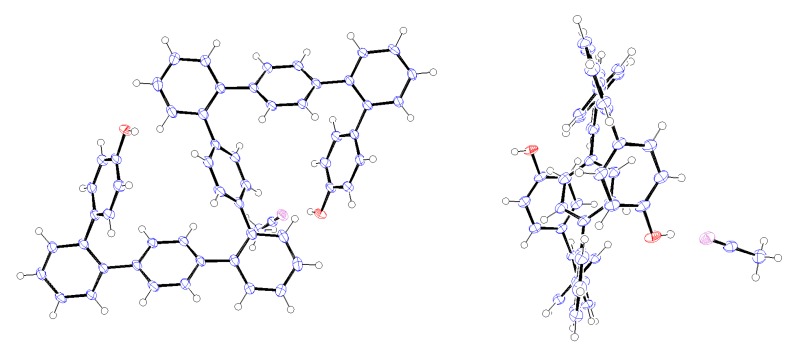
ORTEP representation (50% ellipsoid probability) of the X-ray structure of **12**∙2CH_3_CN. Only one CH_3_CN molecule is shown. Left, front view; Right, side view.

Crystals of 9-mer **12** suitable for X-ray analysis were obtained by recrystallization from CH_3_CN, with the resulting structure shown in Figure 1 [34]. This is the first X-ray structure of *o*,*p*-oligophenylenes that has been obtained. The 9-mer can be seen adopt an S-shaped, centrosymmetric conformation in which the inversion center is located at the central benzene ring. Both of the hydroxy groups were observed to form hydrogen bonds with CH_3_CN molecules.

## 3. Experimental Section

### 3.1. General

All reactions were performed under argon atmosphere. Reactions were monitored by TLC on Merck (Tokyo, Japan) silica gel 60 F_254_ plates visualized by UV lump at 254 nm. Column chromatography was performed on Merck (Tokyo, Japan) silica gel 60 and preparative TLC was performed on Merck (Tokyo, Japan) silica gel 60 F_254_ 0.5 mm plates. NMR spectra were measured on a JEOL (Akishima, Japan) AL-400 NMR spectrometer (400 MHz for ^1^H spectra and 100 MHz for ^13^C spectra) and a JEOL Akishima, Japan) ECA-500 NMR spectrometer (500 MHz for ^1^H spectra and 125 MHz for ^13^C spectra). For ^1^H NMR, tetramethylsilane (TMS) (δ = 0) in CDCl_3_ served as an internal standard. For ^13^C NMR, CDCl_3_ (δ = 77.0) served as an internal standard. Infrared spectra were measured on a SHIMADZU (Kyoto, Japan) IR Prestige-21 spectrometer (ATR). High-resolution mass spectra (HRMS) were measured on a JEOL (Akishima, Japan) JMS-T100TD time-of-flight mass spectrometer (DART), Bruker (Billerica, MA, USA) micrOTOF mass spectrometer (ESI), or Bruker (Billerica, MA, USA) Ultraflex TOF/TOF mass spectrometer (MALDI). Melting points were measured using MPA 100 OptiMelt (Stanford Research Systems, Sunnyvale, CA, USA) and uncorrected. X-ray analysis was made on a Rigaku (Tokyo, Japan) AFC7R diffractometer using graphite monochromated Mo-Kα radiation and a rotating anode generator. 

Anhydrous solvents (except for acetonitrile) were purchased from Kanto Chemical (Tokyo, Japan) and used without further purification. Acetonitrile was purchased from Wako Pure Chemical Industries (Osaka, Japan) and distilled from CaH_2_ under argon. All other chemicals were purchased from Wako Pure Chemical Industries, Kanto Chemical, Tokyo Chemical Industry (Tokyo, Japan), and Aldrich (Milwaukee, US) and used without further purification.

HPLC charts of **5**-**9** and ^1^H- and ^13^C-NMR spectra of **1**, **3**-**14** are shown in Supplementary Materials.

### 3.2. 2''-Hydroxy-[1,1':4',1''-terphenyl]-2-yl) Boronic Acid (**1**)

*n*-BuLi (1.59 M in hexane, 12.6 mL, 20.0 mmol) was added over the course of 3 min to a solution of **2** (3.25 g, 10.0 mmol) in Et_2_O (20.0 mL) at −78 °C under Ar. After 10 min, THF (20.0 mL) was added. After a further 10 min, triisopropyl borate (2.7 mL, 12.0 mmol) was added over the course of 5 min, and the mixture was warmed to room temperature and stirred for 3 h. Aqueous HCl (1 M, 50 mL) was added. The THF was then removed *in vacuo*. The mixture was subsequently extracted with Et_2_O, and the organic layer was washed with brine, dried over Na_2_SO_4_, and concentrated *in vacuo*. The residue was purified by recrystallization (EtOAc/hexane/H_2_O = 10:4:1) to give **1** (2.12 g, 72%) as a white solid. mp. 126.6–129.6 °C; IR (ATR) (cm^−1^) 3500, 1473, 1332, 752; ^1^H-NMR (500 MHz, acetone-*d*_6_ with one drop of D_2_O): δ 6.93 (1H, t, *J* = 7.2 Hz), 7.03 (1H, d, *J* = 8.0 Hz), 7.18 (1H, t, *J* = 8.0 Hz), 7.31–7.37 (2H, m), 7.41–7.44 (2H, m), 7.50 (2H, d, *J* = 8.0 Hz), 7.65 (3H, m); ^13^C-NMR (125 MHz, acetone-*d*_6_ with one drop of D_2_O): δ 116.8, 120.6, 126.8, 129.3, 128.5, 128.9, 129.2, 129.6, 129.8, 131.1, 133.8, 136.3, 138.2, 142.3, 145.9, 155.0; Anal. calcd for C_18_H_15_BO_3_: C, 74.52; H, 5.21, found: C, 74.53; H, 5.27.

### 3.3. 3-mer (OH) (**3**)

[1,1':4',1''-Terphenyl]-2,2''-diol [35] (78.7 mg, 0.300 mmol) and *n*-dodecanoyl chloride (136 mg, 0.622 mmol) were dissolved in TfOH [36] (0.6 mL) at rt, and the mixture was stirred for 1 h at the same temperature. After the reaction was complete, H_2_O (5.0 mL) was added. The mixture was then extracted with CH_2_Cl_2_, and the organic layer was washed with saturated NaHCO_3_ and brine, dried over Na_2_SO_4_, and concentrated *in vacuo*. The residue was purified by recrystallization from methanol to give **3** (160 mg, 85%) as a white solid. mp. 119.4–121.5 °C; IR (ATR) (cm^−1^) 3331, 1467, 1278, 1193, 844, 667; ^1^H-NMR (400 MHz, CDCl_3_): δ 0.87 (6H, t, *J* = 6.8 Hz), 1.26 (32H, bs), 1.74 (4H, quint, *J* = 6.8 Hz), 2.94 (4H, t, *J* = 7.2 Hz), 6.56 (2H, s), 7.06 (2H, d, *J* = 8.4 Hz), 7.62 (4H, s), 7.92 (2H, dd, *J* = 8.8, 2.0 Hz), 7.96 (2H, d, *J* = 2.0 Hz); ^13^C-NMR (100 MHz, CDCl_3_): δ 14.0, 22.6, 24.8, 29.3, 29.45, 29.49, 29.58, 29.6, 31.9, 38.4, 116.2, 127.9, 129.8, 130.0, 130.4, 131.2, 136.4, 157.5, 200.2 (one carbon overlapped.); HRMS (ESI): *m*/*z* calcd for C_42_H_57_O_4_ ([M − H]^−^) 625.4262; found: 625.4275.

### 3.4. 3-mer (ONf) (**4**)

Perfluorobutanesulfonyl fluoride (0.182 mL, 1.02 mmol) was added over 1 min to a solution of **21** (0.160 g, 0.255 mmol) and Et_3_N (0.28 mL, 2.04 mmol) in MeCN (0.85 mL) at 0 °C, and the mixture was stirred for 1 min at the same temperature. The reaction mixture was warmed to room temperature and then stirred for a further 4 h. After the reaction was complete, aqueous HCl (1 M, 5.0 mL) was added. The mixture was extracted with CH_2_Cl_2_, and the organic layer was washed with H_2_O and brine, dried over Na_2_SO_4_, and concentrated *in vacuo*. The residue was purified using silica gel chromatography (hexane/CH_2_Cl_2_ = 4:1) to give **4** (0.250 g, 82%) as a yellow oil. IR (ATR) (cm^−1^) 1695, 1427, 1236, 887; ^1^H-NMR (400 MHz, CDCl_3_): δ 0.86–0.90 (6H, m), 1.28–1.38 (32H, brs), 1.76–1.79 (4H, m), 2.98–3.02 (4H, m), 7.52–7.70 (6H, m), 8.06 (2H, dd, *J* = 2.0, 8.8 Hz), 8.13 (2H, d, *J* = 2.0 Hz); ^13^C-NMR (125 MHz, CDCl_3_): δ 14.1, 22.7, 24.1, 29.26, 29.32, 29.45, 29.48, 29.6, 31.9, 38.8, 122.4, 129.1, 129.7, 131.9, 135.2, 135.5, 137.0, 149.6, 198.6 (one carbon signal overlapped). Nonaflyl carbons were not observed because of the low signal intensities; HRMS (dart): *m*/*z* calcd for C_50_H_57_F_18_O_6_S_2_ ([M + H]^+^) 1191.3202; found: 1191.3194.

### 3.5. 9-mer (OH) (**5**)

Nonaflate **4** (1.23 g, 1.04 mmol), **1** (0.699 g, 2.41 mmol), KF (0.451 g, 7.76 mmol), Pd(OAc)_2_ (23.2 mg, 0.104 mmol), and SPhos (51.0 mg, 0.124 mmol) were placed in a sealable tube, which was then evacuated and backfilled with Ar. A mixture of THF/H_2_O (4:1, 1.0 mL) was then added. The tube was sealed, and the mixture was stirred at 50 °C for 17 h. After the reaction was complete, H_2_O (5.0 mL) was added. The mixture was extracted with CH_2_Cl_2_, and the organic layer was washed with brine, dried over Na_2_SO_4_, and concentrated *in vacuo*. The residue was purified using silica gel chromatography (hexane/CH_2_Cl_2_ = 1:2) to give **5** (0.807 g, 72%) as a white solid. mp. 130.1–134.3 °C; IR (ATR) (cm^−1^) 3516, 1685, 1450, 1267, 827, 746; ^1^H-NMR (400 MHz, CDCl_3_): δ 0.86 (6H, t, *J* = 6.8 Hz), 1.24 (32H, brs), 1.70–1.77 (4H, m), 2.95–3.00 (4H, m), 6.02 (0.5H, s), 6.31 (4.5H, s), 6.53 (3H, d, *J* = 8.0 Hz), 6.57 (1H, s), 6.73 (1H, d, *J* = 7.6 Hz), 6.87–6.97 (4H, m), 7.08–7.39 (16.5H, m), 7.52 (1.5H, d, *J* = 7.6 Hz), 7.85 (2.5H, s), 7.96 (1.5H, d, *J* = 8.0 Hz) (mixture of rotamers); ^13^C-NMR (125 MHz, CDCl_3_): δ 14.1, 22.7, 24.3, 29.31, 29.36, 29.48, 29.5, 29.6, 31.9, 38.7, 115.8, 116.0, 120.7, 120.8, 126.9, 127.0, 127.5, 127.58, 127.6, 127.7, 128.16, 128.2, 128.3, 128.5, 128.9, 129.0, 129.5, 129.7, 129.86, 129.97, 130.0, 130.1, 131.4, 131.5, 132.2, 134.9, 135.1, 135.4, 136.1, 136.2, 138.3, 138.6, 138.8, 139.0, 139.5, 139.9, 140.0, 140.1, 140.8, 140.9, 152.4, 152.6, 200.7 (mixture of rotamers); HRMS (ESI): *m*/*z* calcd for C_78_H_81_O_4_ ([M − H]^−^) 1081.6140; found: 1081.6102.

### 3.6. 9-mer (ONf) (**6**)

Perfluorobutanesulfonyl fluoride (0.70 mL, 3.86 mmol) was added over 1 min to a solution of **5** (1.05 g, 0.965 mmol) and Et_3_N (1.10 mL, 7.72 mmol) in MeCN (3.2 mL) at room temperature, and the mixture was stirred for 2 h at the same temperature. After the reaction was complete, aqueous HCl (1 M, 5.0 mL) was added. The mixture was extracted with CH_2_Cl_2_, and the organic layer was washed with H_2_O and brine, dried over Na_2_SO_4_, and concentrated *in vacuo*. The residue was purified using silica gel chromatography (hexane/CH_2_Cl_2_ = 3:1) to give **6** (1.46 g, 92%) as a colorless oil. IR (ATR) (cm^−1^) 1685, 1236, 1142, 835, 765; ^1^H-NMR (500 MHz, CDCl_3_): δ 0.87 (6H, t, *J* = 7.5 Hz), 1.25–1.32 (32H, m), 1.68–1.73 (4H, m), 2.92–2.98 (4H, m), 6.44 (2.6H, s), 6.648 (2.6H, d, *J* = 8.5 Hz), 6.649 (1.4H, s), 6.80 (1.4H, d, *J* = 8.5 Hz), 7.09 (2.6H, d, *J* = 8.5 Hz), 7.12 (1.4H, d, *J* = 8.0 Hz), 7.29–7.51 (18H, m), 7.70 (1.3H, s), 7.83 (0.7H, s), 7.86 (0.7H, d, *J* = 8.0 Hz), 7.95 (1.3H, dd, *J* = 8.0, 1.5 Hz) (mixture of rotamers); ^13^C-NMR (100 MHz, CDCl_3_): δ 14.1, 22.8, 24.3, 24.4, 29.4, 29.60, 29.63, 29.7, 32.0, 38.6, 121.9, 126.6, 127.5, 128.1, 128.2, 128.56, 128.63, 128.7, 128.88, 128.9, 129.0, 129.7, 129.9, 130.1, 130.2, 130.3, 131.2, 131.5, 131.9, 132.1, 133.9, 135.7, 136.2, 136.3, 138.9, 139.2, 139.5, 140.35, 140.43, 140.8, 140.9, 141.2, 141.4, 141.47, 144.54, 147.3, 200.2, 200.3 (mixture of rotamers) (Nonaflyl carbons were not observed because of the low signal intensities) HRMS (MALDI, α-cyano-4-hydroxycinnamic acid as the matrix): *m*/*z* calcd for C_86_H_80_F_18_O_6_S_2_ ([M + H]^+^) 1646.5007; found: 1647.5108.

### 3.7. 15-mer (OH) (**7**)

Nonaflate **6** (0.972 g, 0.590 mmol), **1** (0.514 g, 1.77 mmol), KF (0.257 g, 4.43 mmol), Pd(OAc)_2_ (13.2 mg, 0.0590 mmol), and SPhos (29.1 mg, 0.0708 mmol) were placed in a flask, which was then evacuated and backfilled with Ar. A mixture of THF/H_2_O (4:1, 0.59 mL) was then added. The tube was sealed, and the mixture was stirred at 70 °C for 17 h. After the reaction was complete, H_2_O (5.0 mL) was added. The mixture was extracted with CH_2_Cl_2_, and the organic layer was washed with brine, dried over Na_2_SO_4_, and concentrated *in vacuo*. The residue was purified using silica gel chromatography (hexane/EtOAc = 10:1) to give **7** (0.711 g, 78%) as a yellow solid. mp. 109.5–115.0 °C; IR (ATR) (cm^−1^) 3529, 1678, 1467, 1269, 829, 748; ^1^H-NMR (500 MHz, CDCl_3_): δ 0.87 (6H, m), 1.24 (32H, brs), 1.71 (4H, brs), 2.95 (4H, brs), 5.50–6.70 (18H, m), 6.92–7.97 (42H, m) (mixture of rotamers); ^13^C-NMR (100 MHz, CDCl_3_): δ 14.1, 22.7, 24.2, 24.29, 29.31, 29.35, 29.38, 29.48, 29.51, 29.6, 31.9, 38.7, 115.7, 115.8, 120.5, 120.6, 120.7, 126.9, 127.3, 127.5, 127.7, 127.9, 128.0, 128.3, 128.4, 128.6, 128.7, 128.9, 129.0, 129.6, 129.8, 129.9, 131.4, 131.6, 134.8, 136.0, 138.26, 138.30, 138.4, 138.5, 138.9, 139.0, 139.7, 140.0, 140.1, 140.2, 140.3, 140.8, 141.6, 152.7, 152.8, 200.4 (mixture of rotamers); HRMS (ESI): *m*/*z* calcd for C_114_H_105_O_4_ ([M − H]^−^) 1537.8018; found: 1537.8010.

### 3.8. 15-mer (ONf) (**8**)

Perfluorobutanesulfonyl fluoride (0.11 mL, 0.604 mmol) was added over 1 min to a solution of **7** (0.233 g, 0.151 mmol) and Et_3_N (0.17 mL, 1.21 mmol) in MeCN (0.5 mL) at room temperature, and the mixture was stirred for 2 h at the same temperature. After the reaction was complete, aqueous HCl (1 M, 10.0 mL) was added. The mixture was extracted with CH_2_Cl_2_, and the organic layer was washed with H_2_O and brine, dried over Na_2_SO_4_, and concentrated *in vacuo*. The residue was purified using silica gel chromatography (hexane/CH_2_Cl_2_ = 4:1) to give **8** (0.146 g, 46%) as a colorless oil. IR (ATR) (cm^−1^) 1685, 1423, 1236, 889, 732; ^1^H-NMR (400 MHz, CDCl_3_): δ 0.85–0.88 (6H, m), 1.25 (32H, brs), 1.71 (4H, brs), 2.95 (4H, brs), 5.65–6.71 (18H, m), 6.91–7.95 (42H, m) (mixture of rotamers); ^13^C-NMR (100 MHz, CDCl_3_): δ 14.1, 22.7, 24.2, 24.3, 29.3, 29.4, 29.5, 29.6, 31.9, 38.6, 118.5, 121.9, 126.4, 126.8, 127.1, 127.4, 127.9, 128.2, 128.5, 128.6, 128.75, 128.83, 128.9, 129.1, 129.4, 129.8, 129.9, 130.0, 130.2, 130.7, 131.3, 131.7, 131.8, 132.1, 133.4, 133.5, 135.5, 135.6, 136.1, 138.5, 138.6, 139.1, 139.3, 139.6, 140.1, 140.3, 141.0, 141.1, 141.6, 144.9, 145.2, 147.0, 199.7, 200.2 (mixture of rotamers) (Nonaflyl carbons were not observed because of the low signal intensities); HRMS (MALDI, α-cyano-4-hydroxycinnamic acid as a matrix): *m*/*z* calcd for C_122_H_105_F_18_O_6_S_2_ ([M + H]^+^) 2103.6958; found: 2103.7120.

### 3.9. 21-mer (OH) (**9**)

Nonaflate **8** (0.186 g, 0.088 mmol), **1** (76.9 mg, 0.264 mmol), KF (38.3 mg, 0.660 mmol), Pd(OAc)_2_ (2.0 mg, 0.00880 mmol), and SPhos (4.3 mg, 0.0106 mmol) were placed in a flask, which was then evacuated and backfilled with Ar. A mixture of THF/H_2_O (4:1, 0.09 mL) was then added. The tube was sealed, and the mixture was stirred at 70 °C for 17 h. After the reaction was complete, H_2_O (5.0 mL) was added. The mixture was extracted with CH_2_Cl_2_, and the organic layer was washed with brine, dried over Na_2_SO_4_, and concentrated *in vacuo*. The residue was purified using silica gel chromatography (hexane/CH_2_Cl_2_ = 1:2) to give **9** (76.3 mg, 43%) as a white solid. mp. 135.3–138.9 °C; IR (ATR) (cm^−1^) 3537, 1678, 1467, 748; ^1^H-NMR (500 MHz, CDCl_3_): δ 0.85–0.86 (6H, m), 1.24 (32H, bs), 1.49–1.72 (4H, m), 2.73–2.91 (4H, m), 5.35–5.60 (2H, m), 6.04–6.67 (23H, m), 6.83–8.00 (59H, m) (mixture of rotamers); ^13^C-NMR (100 MHz, CDCl_3_): δ 14.1, 22.7, 23.9, 24.2, 29.28, 29.33, 29.5, 29.6, 29.7, 31.9, 38.4, 38.6, 115.7, 120.7, 126.4, 126.8, 126.9, 127.1, 127.3, 127.4, 127.5, 127.6, 127.7, 127.96, 128.04, 128.2, 128.5, 128.6, 128.9, 129.0, 129.7, 130.0, 130.3, 130.7, 131.1, 131.3, 131.4, 134.4, 135.9, 138.37, 138.45, 138.6, 138.8, 138.9, 139.1, 139.3, 139.6, 139.8, 139.9, 140.0, 140.1, 140.2, 140.4, 140.9, 141.0, 141.4, 144.9, 152.5, 152.6, 151.7, 199.5, 200.1 (mixture of rotamers); HRMS (ESI): *m*/*z* calcd for C_150_H_129_O_4_ ([M − H]^−^) 1993.9896; found: 1993.9873.

### 3.10. 7-mer (OH) (**10**)

4-Dibromobenzene (236.5 mg, 1.00 mmol), **1** (725.6 mg, 2.50 mmol), KF (437.2 mg, 7.53 mmol), Pd(OAc)_2_ (22.7 mg, 0.101 mmol), and SPhos (49.7 mg, 0.121 mmol) were placed in a sealable tube, which was then evacuated and backfilled with Ar. A mixture of THF/H_2_O (4:1, 1.0 mL) was then added. The tube was sealed, and the mixture was stirred at 70 °C for 21 h. After the reaction was complete, H_2_O (10.0 mL) was added. The mixture was extracted with EtOAc, and the organic layer was washed with brine, dried over MgSO_4_, and concentrated *in vacuo*. The residue was purified by recrystallization (toluene) to give **10** (412.4 mg, 72%) as a white solid. mp. 255.6–257.5 °C; IR (ATR) (cm^−1^) 3003, 1711, 1358, 1221, 750; ^1^H-NMR (400 MHz, CDCl_3_) δ: 5.14 (2H, s), 6.93 (2H, d, *J* = 7.8 Hz), 6.95 (2H, d, *J* = 7.8 Hz), 7.07 (4H, s), 7.20–7.23 (2H, m), 7.26 (4H, d, *J* = 8.3 Hz), 7.33 (4H, d, *J* = 8.3 Hz), 7.41–7.46 (10H, m); ^13^C-NMR (125 MHz, CDCl_3_) δ: 115.9, 121.0, 127.7, 127.8, 128.5, 129.2, 129.6, 130.3, 130.6, 130.8, 130.8, 135.3, 139.8, 139.9, 140.3, 141.2, 152.5; HRMS (ESI): *m*/*z* calcd for C_42_H_29_O_2_ ([M − H]^−^) 565.2173; found: 565.2170.

### 3.11. 7-mer (ONf) (**11**)

Perfluorobutanesulfonyl fluoride (1.65 mL, 9.40 mmol) was added over 1 min to a solution of **10** (1.31 g, 2.31 mmol) and Et_3_N (2.6 mL, 18.7 mmol) in MeCN (7.9 mL) at room temperature, and the mixture was stirred for 2 h at the same temperature. After the reaction was complete, aqueous HCl (1 M, 20.0 mL) was added. The mixture was extracted with EtOAc, and the organic layer was washed with H_2_O and brine, dried over MgSO_4_, and concentrated *in vacuo*. MeOH was then added to the residue. After stirring, filtration gave **11** (1.65 g, 63%) as a white solid. mp. 198.5–199.8 °C; IR (ATR) (cm^−1^) 1429, 1202, 1140; ^1^H-NMR (400 MHz, CDCl_3_) δ: 7.03 (4H, s), 7.24 (4H, d, *J* = 8.3 Hz), 7.31 (4H, d, *J* = 8.3 Hz), 7.35–7.45 (16H, m); ^13^C-NMR (125 MHz, CDCl_3_) δ: 121.9, 127.4, 127.7, 128.6, 128.9, 129.7, 130.2, 130.5, 130.7, 132.0, 133.9, 135.9, 139.4, 139.9, 140.5, 141.9, 147.3; HRMS (ESI): *m/z* calcd for C_50_H_28_F_18_NaO_6_S_2_ ([M + Na]^+^) 1153.0932; found: 1153.0875.

### 3.12. 9-mer (OH) (**12**)

Nonaflate **11** (564 mg, 0.499 mmol), 4-hydroxyphenylboronic acid (173.6 mg, 1.26 mmol), KF (219.3 mg, 3.78 mmol), Pd(OAc)_2_ (11.8 mg, 0.0526 mmol), and SPhos (25.1 mg, 0.0611 mmol) were placed in a sealable tube, which was then evacuated and backfilled with Ar. A mixture of THF/H_2_O (4:1, 1.0 mL) was then added. The tube was sealed, and the mixture was stirred at 70 °C for 23 h. H_2_O (10.0 mL) was added. The mixture was extracted with EtOAc, and the organic layer was washed with brine, dried over MgSO_4_, and concentrated *in vacuo*. The residue was purified using silica gel chromatography (hexane/EtOAc = 4:1 to 2:1) to give **12** (222.2 mg, 62%) as a white solid. mp. 244.1–246.5 °C; IR (ATR) (cm^−1^) 3003, 1709, 1358, 1219, 1092, 903; ^1^H-NMR (400 MHz, CDCl_3_) δ: 3.82 (2H, s), 6.39 (4H, d, *J* = 8.8 Hz), 6.85 (4H, d, *J* = 8.8 Hz), 6.87 (4H, d, *J* = 8.3 Hz), 6.93 (4H, d, *J* = 8.3 Hz), 6.93 (4H, s), 7.32–7.52 (16H, m); ^13^C-NMR (125 MHz, CDCl_3_) δ: 114.9, 127.2, 127.4, 127.6, 127.7, 129.2, 129.6, 129.7, 130.4, 130.5, 130.6, 130.6, 131.0, 139.2, 139.4, 140.1, 140.3, 140.4, 140.5, 140.5, 154.1; HRMS (ESI): *m*/*z* calcd for C_54_H_37_O_2_ ([M − H]^−^) 717.2799; found: 717.2770.

### 3.13. 9-mer (ONf) (**13**)

Perfluorobutanesulfonyl fluoride (0.21 mL, 1.20 mmol) was added over 1 min to a solution of **12** (216.4 mg, 0.301 mmol) and Et_3_N (0.33 mL, 2.37 mmol) in MeCN/CH_2_Cl_2_ (1:1, 1.0 mL) at room temperature, and the mixture was stirred for 22 h at the same temperature. After the reaction was complete, aqueous HCl (1 M, 5.0 mL) was added. The mixture was extracted with EtOAc, and the organic layer was washed with H_2_O and brine, dried over MgSO_4_, and concentrated *in vacuo*. MeOH was then added to the residue. After stirring, filtration gave **13** (298.4 mg, 77%) as a white solid. mp. 204.4–205.8 °C; IR (ATR) (cm^−1^) 1713, 1429, 1358, 1221, 1138, 845; ^1^H-NMR (400 MHz, CDCl_3_) δ: 6.89 (4H, d, *J* = 8.3 Hz), 6.94 (4H, d, *J* = 8.3 Hz), 6.95 (4H, s), 7.01 (4H, d, *J* = 8.8 Hz), 7.10 (4H, d, *J* = 8.8 Hz), 7.33–7.43 (16H, m); ^13^C-NMR (125 MHz, CDCl_3_) δ: 120.8, 127.7, 127.7, 128.3, 129.3, 129.5, 129.7, 130.4, 130.5, 130.5, 130.6, 131.6, 138.6, 139.1, 139.8, 139.9, 140.1, 140.2, 140.4, 142.1, 148.6; HRMS (ESI): *m*/*z* calcd for C_62_H_36_ClF_18_O_6_S_2_ ([M + Cl]^−^) 1317.1360; found: 1317.1356.

### 3.14. 15-mer (OH) (**14**)

Nonaflate **13** (320.5 mg, 0.250 mmol), **1** (218.6 mg, 0.753 mmol), K_3_PO_4_∙nH_2_O (502.2 mg), Pd(OAc)_2_ (5.6 mg, 0.0249 mmol), and SPhos (20.5 mg, 0.0499 mmol) were placed in a sealable tube, which was then evacuated and backfilled with Ar. Toluene (2.0 mL) was then added. The tube was sealed, and the mixture was stirred at 120 °C for 24 h. After the reaction was complete, H_2_O (10.0 mL) was added. The mixture was extracted with EtOAc and CHCl_3_, and the organic layer was washed with brine, dried over MgSO_4_, and concentrated *in vacuo*. The residue was purified using silica gel chromatography (hexane/CH_2_Cl_2_ = 1:1 to 1:2) to give **14** (126.3 mg, 43%) as a white solid. mp. 280.2–282.0 °C; IR (ATR) (cm^−1^) 3003, 1709, 1358, 1219, 1092, 903; ^1^H-NMR (400 MHz, CDCl_3_) δ: 5.03 (2H, s), 6.88 (2H, d, *J* = 8.3 Hz), 6.90 (4H, s), 6.91 (8H, s), 6.94 (8H, s), 7.11 (2H, dd, *J* = 7.8, 2.0 Hz), 7.16–7.19 (6H, m), 7.21–7.24 (4H, m), 7.27–7.41 (26H, m); ^13^C-NMR (125 MHz, CDCl_3_) δ: 115.9, 120.9, 127.5, 127.5, 127.6, 127.8, 128.5, 129.1, 129.4, 129.5, 129.6, 130.3, 130.5, 130.6, 130.7, 130.8, 135.2, 139.6, 139.7, 139.8, 139.9, 140.2, 140.3, 140.4, 141.1, 152.5, 153.2; HRMS (ESI): *m*/*z* calcd for C_90_H_61_O_2_ ([M − H]^−^) 1173.4677; found: 1173.4636.

## 4. Conclusions

In conclusion, a repetitive two-step method for oligophenylene synthesis using hydroxyterphenylboronic acid **1** has been developed. By employing this method, *o*,*o*,*p*-oligophenylenes with precise chain lengths were readily synthesized. Furthermore, the combined use of **1** and 4-hydroxyphenylboronic acid efficiently gave *o*,*p*-oligophenylenes. The X-ray structure of 9-mer **12** was also determined. The synthetic strategy presented here is applicable to oligophenylenes with various connectivity patterns. By introducing a substituent on the benzene rings of the boronic acids, it would be also possible to synthesize oligophenylenes with substituents at a desired position. The present work not only contributes to the progress of oligophenylene chemistry, but also extends the applicability of Pd-catalyzed cross coupling. 

## Supplementary Materials

Supplementary materials can be accessed at: http://www.mdpi.com/1420-3049/18/12/15207/s1.
