# Supplementary material for: Repetitive Two-Step Method for o,o,p- and o,p-Oligophenylene Synthesis through Pd-Catalyzed Cross-Coupling of Hydroxyterphenylboronic Acid
Source: Molecules. 2013 Dec 10;18(12):15207–19. doi: 10.3390/molecules181215207 (PMC6270598; doi:10.3390/molecules181215207)
Supplement: Supplementary file 1 [file molecules-18-15207-s001.pdf]

Supporting Information

Repetitive Two-Step Method for *o,o,p-* and *o,p-*Oligophenylene Synthesis through Pd-Catalyzed Cross-Coupling of Hydroxyterphenylboronic Acid

Miyuki Yamaguchi, Takeshi Kimura, Naomi Shinohara, and Kei Manabe *

Table of Contents **Pages**

HPLC Charts	S2–S6
NMR Spectra	S7–S19

HPLC Charts

For compounds that showed complicated NMR spectra due to the presence of rotamers, HPLC analysis was performed to confirm their purity.

5

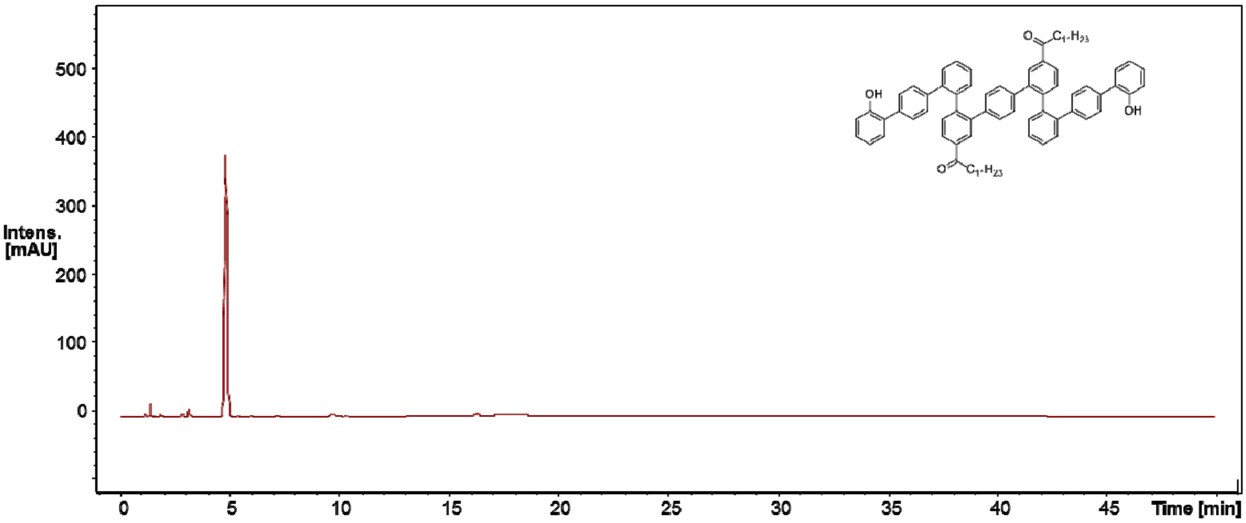

【HPLC method】
Analysis equipment: Agilent Technologies 1200 series
Column: Eclipse XDB-C18 (4.6 × 150mm)
Solvent A: 10% CH_3COONH_3aq, Solvent B : CH_3CN
Gradient (B): 0-15 min, 20 %, 15-40 min, 20-5 %, 40-40.1min, 5-20%
Oven column: 40℃
Injection: 10μl
Flow rate: 0.8mL/min
Detection: 254nm

6

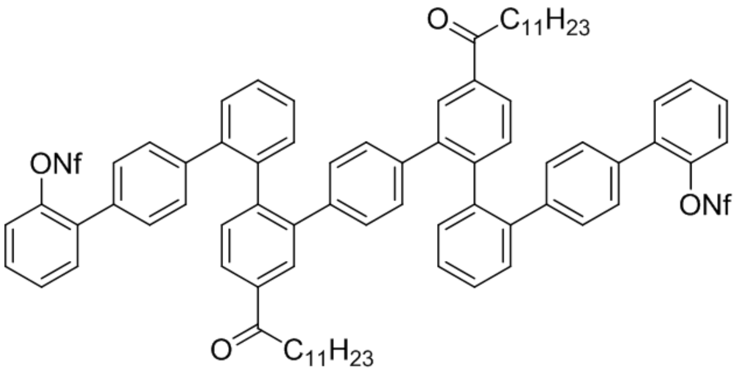

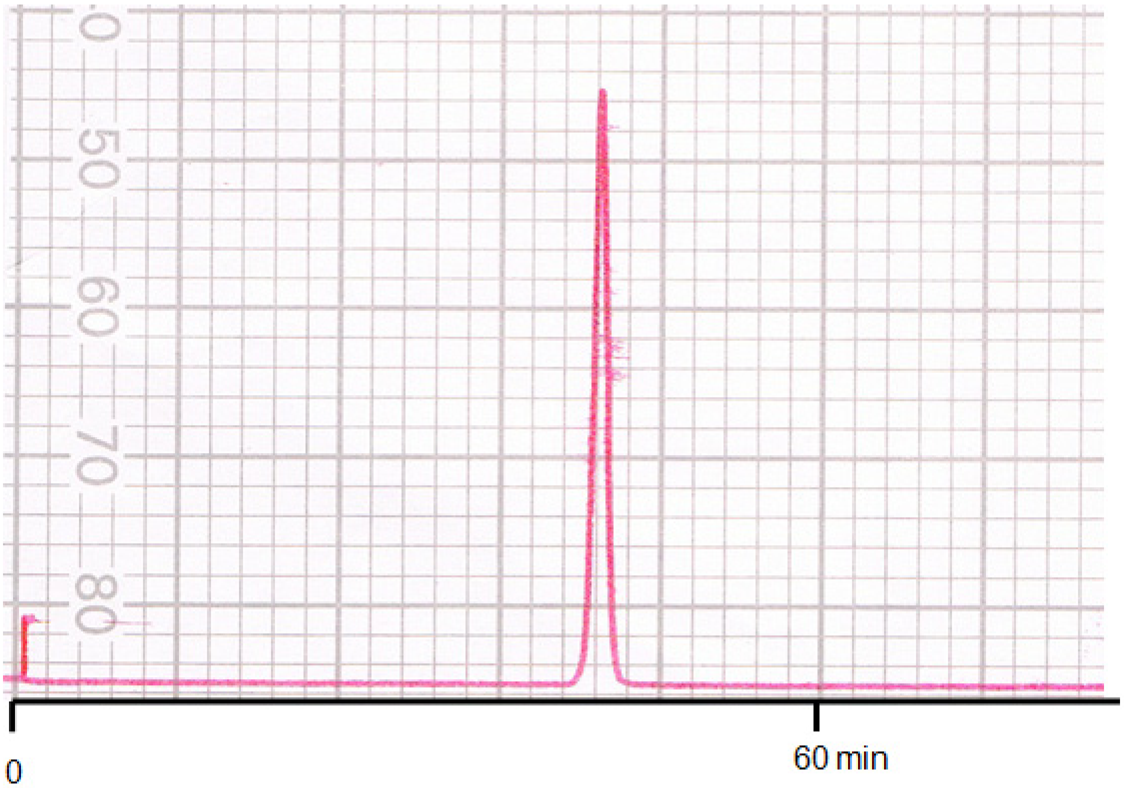

[HPLC method] (gel permeation chromatography)
Analysis equipment: LC-9201 (Japan Analytical Industry)
Column: JALGEL-1H, 2H
Solvent: $CHCl_3$
Flow rate: 3.5 mL/min
Detection: 254 nm

7

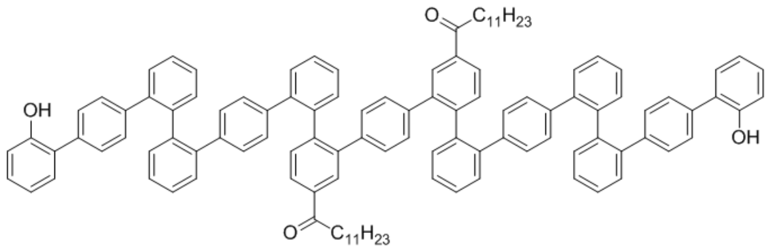

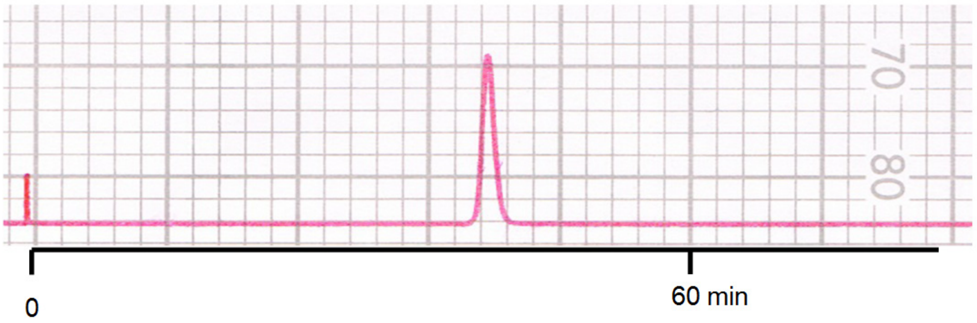

[HPLC method] (gel permeation chromatography)
Analysis equipment: LC-9201 (Japan Analytical Industry)
Column: JALGEL-1H, 2H
Solvent: $CHCl_3$
Flow rate: 3.5 mL/min
Detection: 254 nm

8

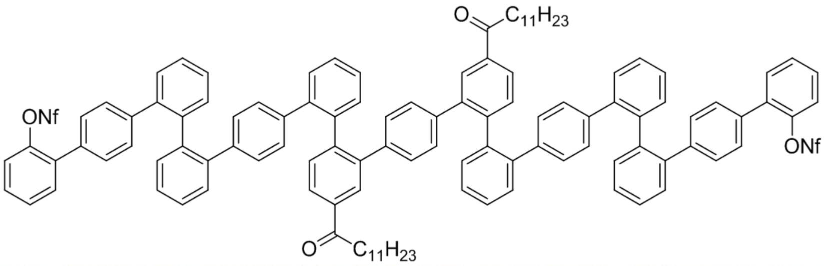

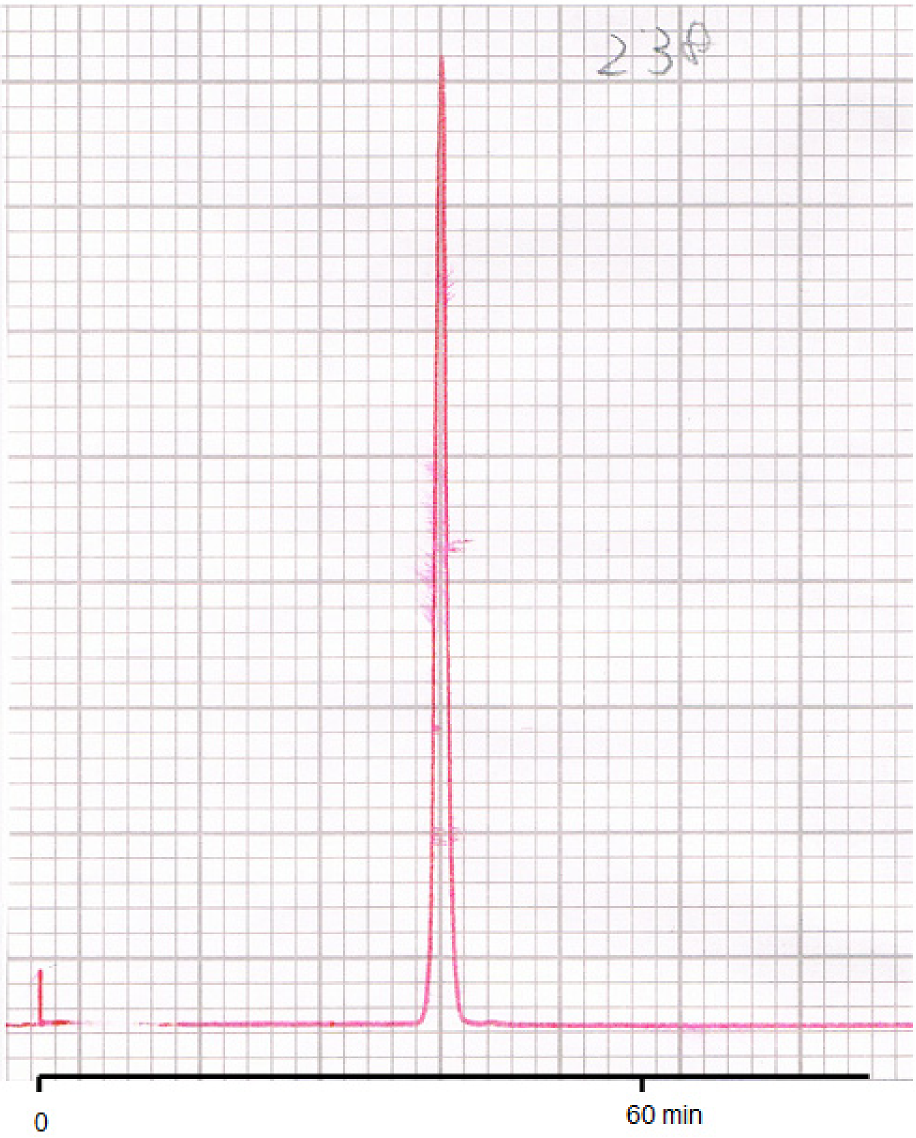

[HPLC method] (gel permeation chromatography)
Analysis equipment: LC-9201 (Japan Analytical Industry)
Column: JALGEL-1H, 2H
Solvent: $CHCl_3$
Flow rate: 3.5 mL/min
Detection: 254 nm

9

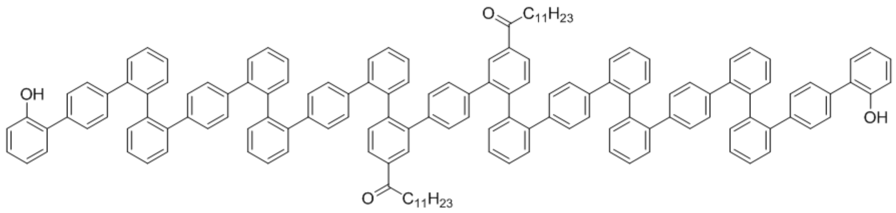

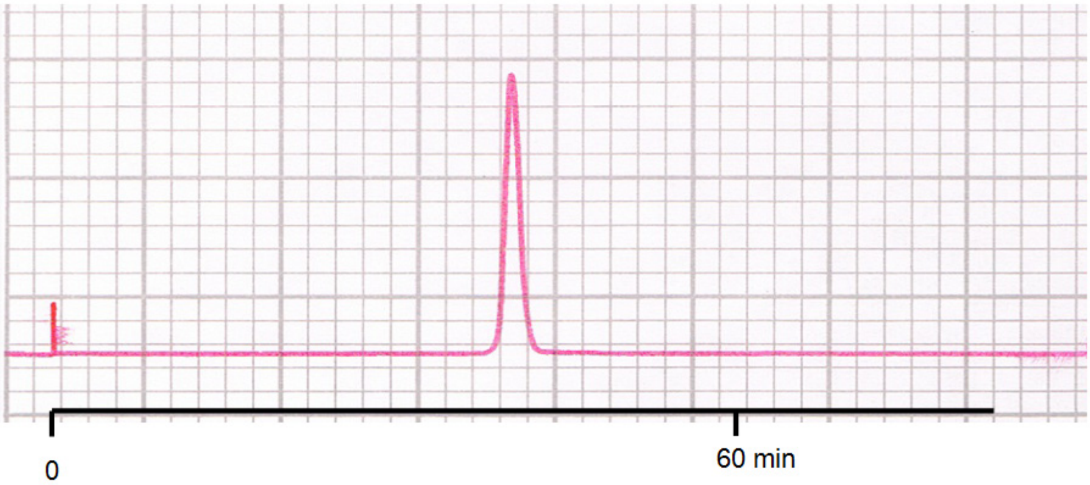

[HPLC method] (gel permeation chromatography)
Analysis equipment: LC-9201 (Japan Analytical Industry)
Column: JALGEL-1H, 2H
Solvent: $CHCl_3$
Flow rate: 3.5 mL/min
Detection: 254 nm

NMR Spectra

1

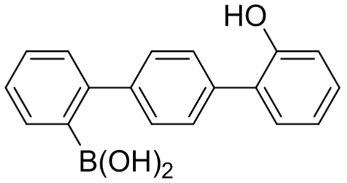

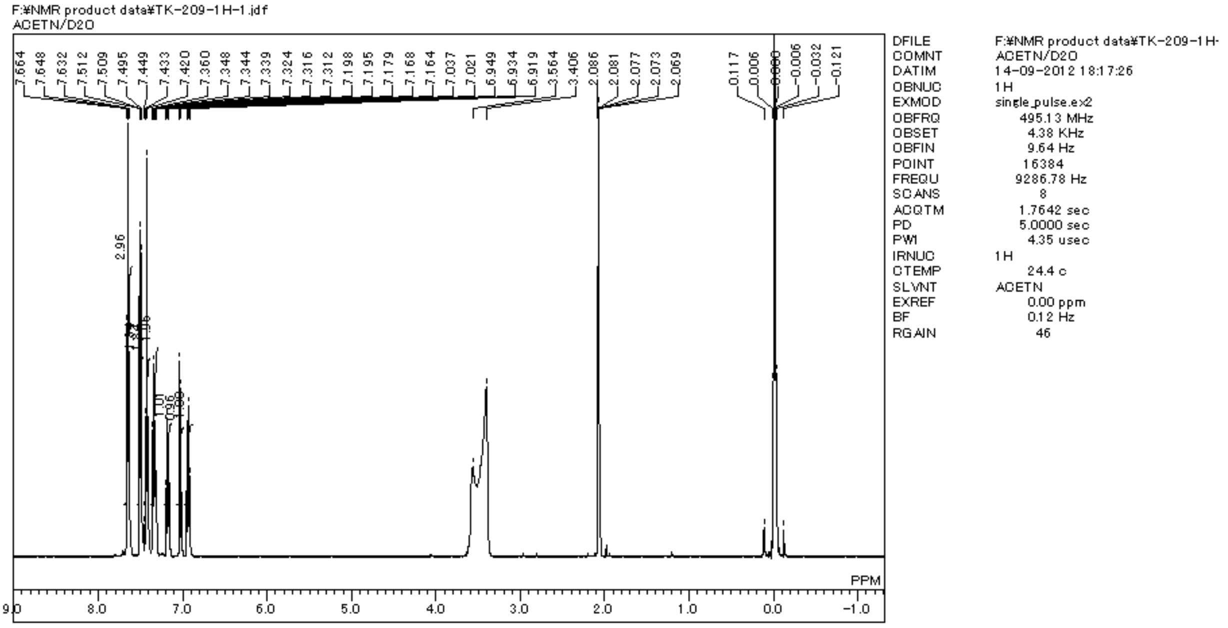

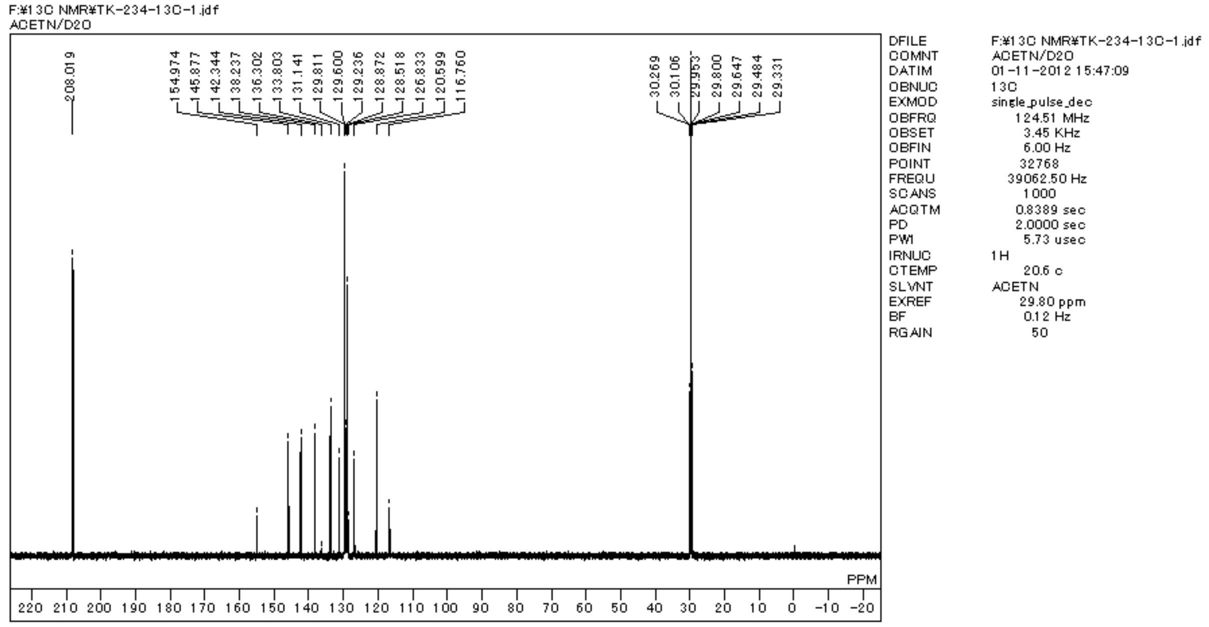

3

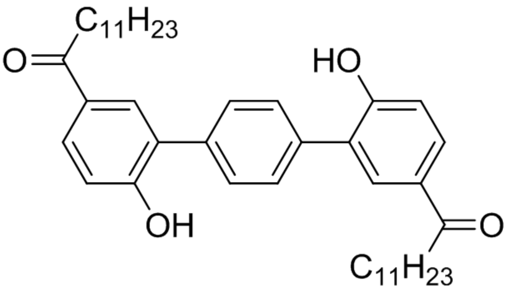
C11H23
O
HO
OH
O
C11H23

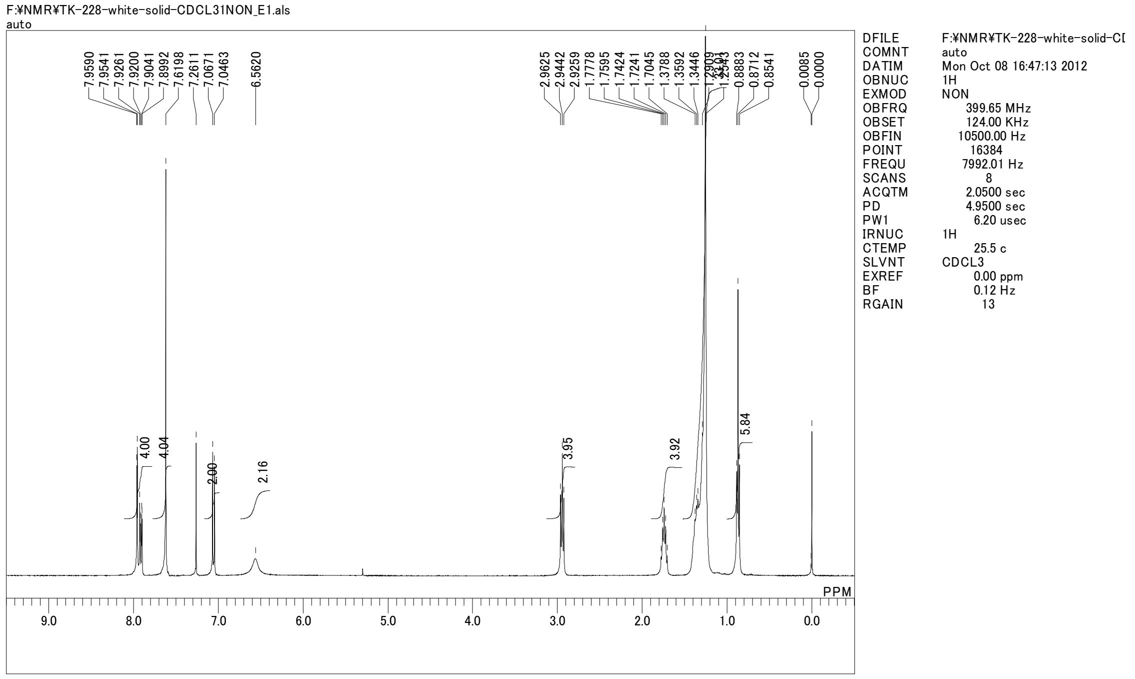
F:¥NMR¥TK-228-white-solid-CDCL31NON_E1.als
auto
7.9590
7.9541
7.9261
7.9200
7.9041
7.8992
7.6198
7.2611
7.0671
7.0463
6.5620
2.9625
2.9442
2.9259
1.7778
1.7595
1.7424
1.7241
1.7045
1.3788
1.3592
1.3446
0.8883
0.8712
0.8541
0.0085
0.0000
4.00
4.04
2.00
2.16
3.95
3.92
5.84
PPM
9.0
8.0
7.0
6.0
5.0
4.0
3.0
2.0
1.0
0.0
DFILE F:¥NMR¥TK-228-white-solid-CI
COMNT auto
DATIM Mon Oct 08 16:47:13 2012
OBNUC 1H
EXMOD NON
OBFRQ 399.65 MHz
OBSET 124.00 KHz
OBFIN 10500.00 Hz
POINT 16384
FREQU 7992.01 Hz
SCANS 8
ACQTM 2.0500 sec
PD 4.9500 sec
PW1 6.20 usec
IRNUC 1H
CTEMP 25.5 c
SLVNT CDCL3
EXREF 0.00 ppm
BF 0.12 Hz
RGAIN 13

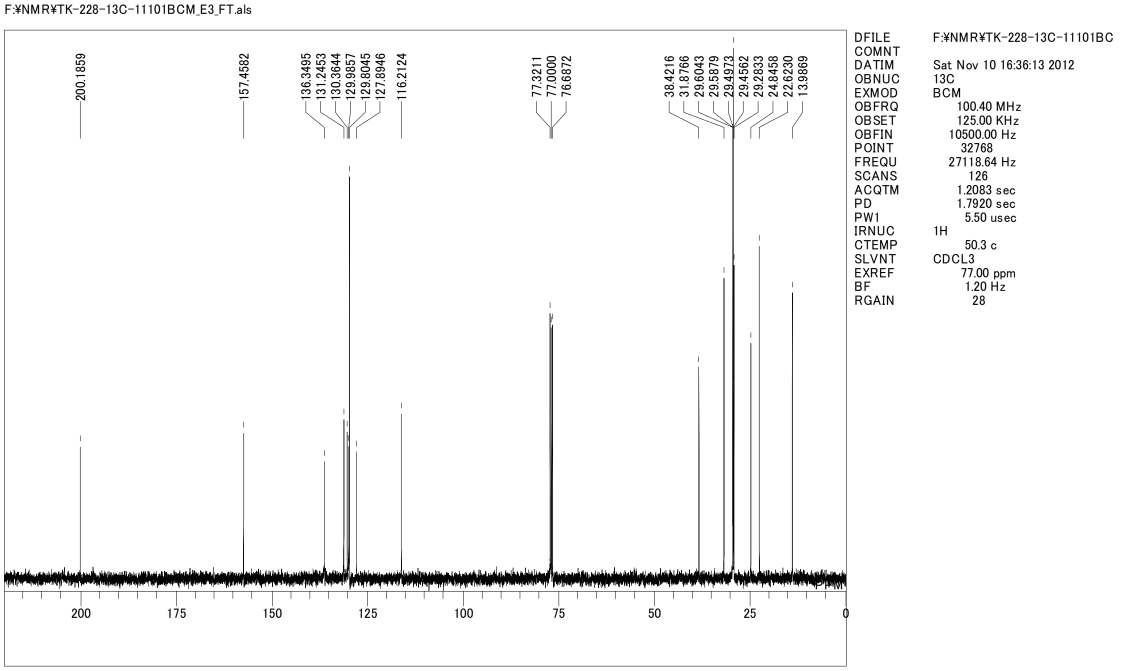
F:¥NMR¥TK-228-13C-11101BCM_E3_FT.als
200.1859
157.4582
136.3495
131.2453
130.3644
129.9857
129.8045
127.8946
116.2124
77.3211
77.0000
76.6872
38.4216
31.8766
29.6043
29.5879
29.4973
29.4562
29.2833
24.8458
22.6230
13.9869
PPM
200
175
150
125
100
75
50
25
0
DFILE F:¥NMR¥TK-228-13C-11101BC
COMNT
DATIM Sat Nov 10 16:36:13 2012
OBNUC 13C
EXMOD BCM
OBFRQ 100.40 MHz
OBSET 125.00 KHz
OBFIN 10500.00 Hz
POINT 32768
FREQU 27118.64 Hz
SCANS 126
ACQTM 1.2083 sec
PD 1.7920 sec
PW1 5.50 usec
IRNUC 1H
CTEMP 50.3 c
SLVNT CDCL3
EXREF 77.00 ppm
BF 1.20 Hz
RGAIN 28

4

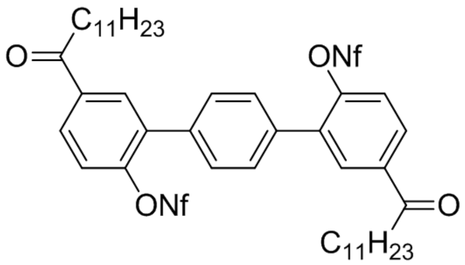
C11H23
O
ONf
ONf
O
C11H23

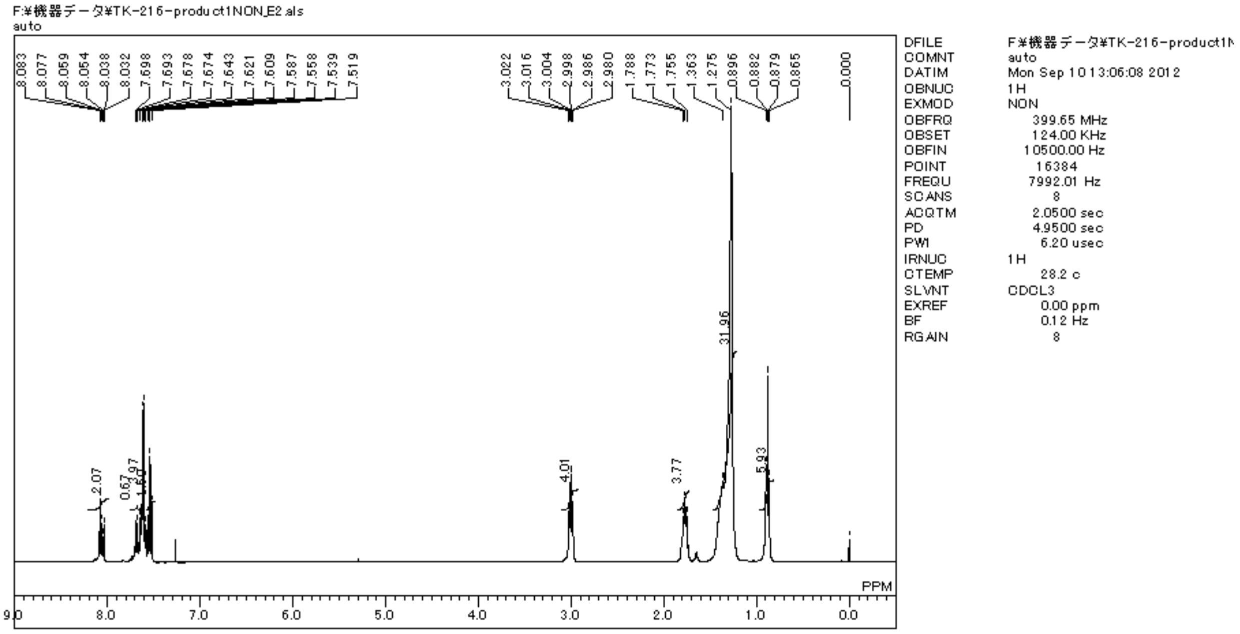
F:¥機器データ¥TK-216-product1NON_E2.als
auto
8.083 8.077 8.059 8.054 8.038 8.032 7.698 7.693 7.678 7.674 7.643 7.621 7.609 7.587 7.558 7.539 7.519
3.022 3.016 3.004 2.998 2.986 2.980 1.788 1.773 1.755 1.363 1.275 0.896 0.882 0.879 0.865 0.000
2.07 0.67 1.97 4.01 3.77 31.96 5.93
PPM
9.0 8.0 7.0 6.0 5.0 4.0 3.0 2.0 1.0 0.0
DFILE F:¥機器データ¥TK-216-product1N
COMNT auto
DATIM Mon Sep 10 13:06:08 2012
OBNUC 1H
EXMOD NON
OBFRQ 399.65 MHz
OBSET 124.00 KHz
OBFIN 10500.00 Hz
POINT 16384
FREQU 7992.01 Hz
SCANS 8
ACQTM 2.0500 sec
PD 4.9500 sec
PW1 6.20 usec
IRNUC 1H
CTEMP 28.2 c
SLVNT CDCL3
EXREF 0.00 ppm
BF 0.12 Hz
RGAIN 8

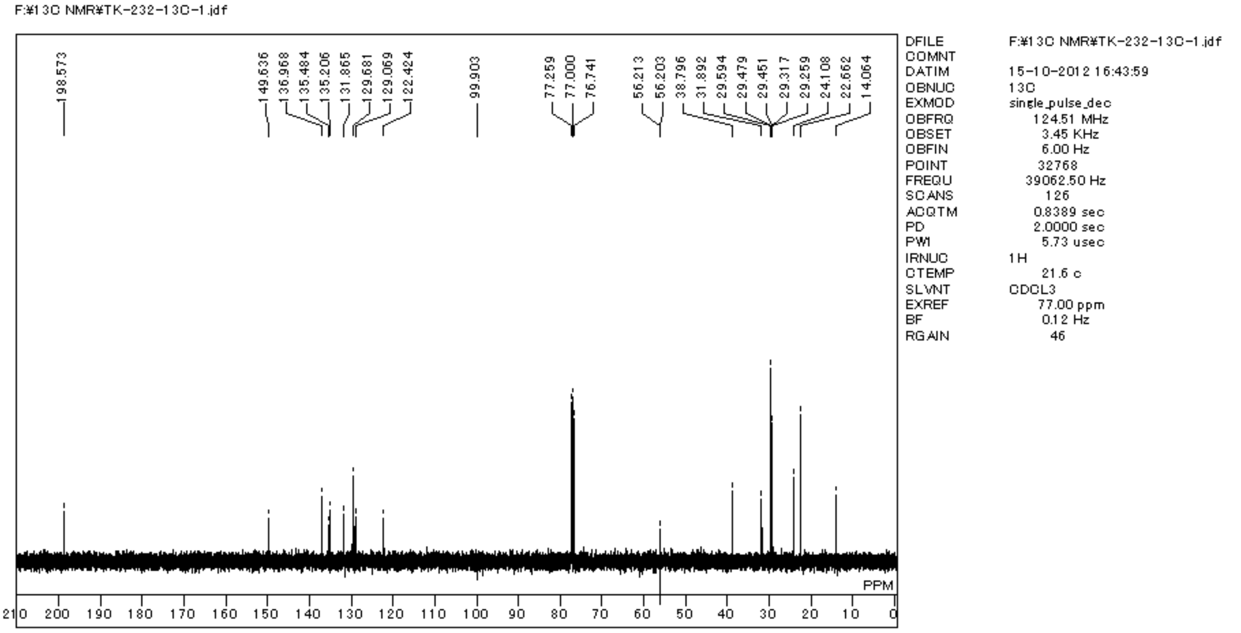
F:¥13C NMR¥TK-232-13C-1.jdf
198.573
149.636 136.968 135.484 135.206 131.865 129.681 129.069 122.424
99.903
77.259 77.000 76.741
56.213 56.203 38.796 31.892 29.594 29.479 29.451 29.317 29.259 24.108 22.662 14.064
PPM
210 200 190 180 170 160 150 140 130 120 110 100 90 80 70 60 50 40 30 20 10 0
DFILE F:¥13C NMR¥TK-232-13C-1.jdf
COMNT
DATIM 15-10-2012 16:43:59
OBNUC 13C
EXMOD single_pulse_dec
OBFRQ 124.51 MHz
OBSET 3.45 KHz
OBFIN 6.00 Hz
POINT 32768
FREQU 39062.50 Hz
SCANS 126
ACQTM 0.8389 sec
PD 2.0000 sec
PW1 5.73 usec
IRNUC 1H
CTEMP 21.6 c
SLVNT CDCL3
EXREF 77.00 ppm
BF 0.12 Hz
RGAIN 46

5

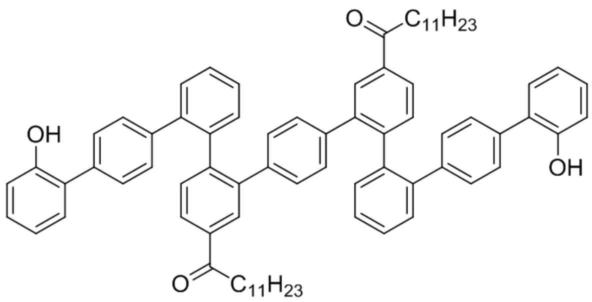
O
$C_{11}H_{23}$
OH
OH
O
$C_{11}H_{23}$

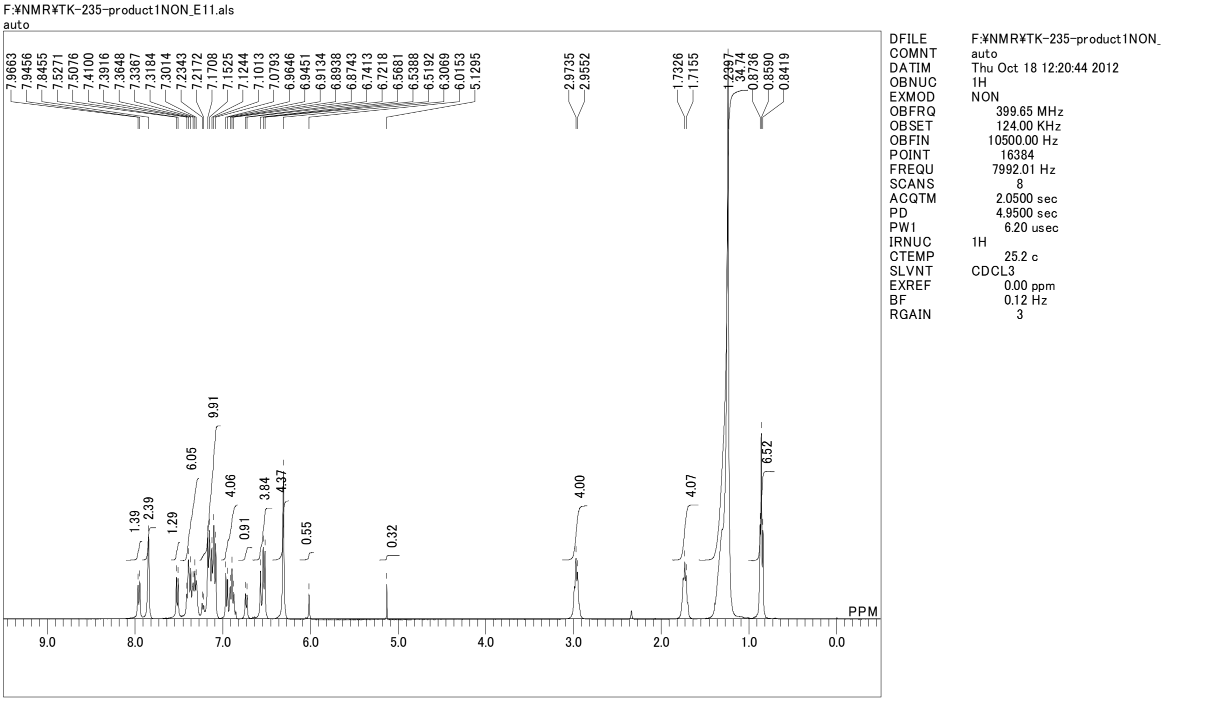
F:¥NMR¥TK-235-product1NON_E11.als
auto
DFILE F:¥NMR¥TK-235-product1NON_
COMNT auto
DATIM Thu Oct 18 12:20:44 2012
OBNUC 1H
EXMOD NON
OBFRQ 399.65 MHz
OBSET 124.00 KHz
OBFIN 10500.00 Hz
POINT 16384
FREQU 7992.01 Hz
SCANS 8
ACQTM 2.0500 sec
PD 4.9500 sec
PW1 6.20 usec
IRNUC 1H
CTEMP 25.2 c
SLVNT CDCL3
EXREF 0.00 ppm
BF 0.12 Hz
RGAIN 3
PPM

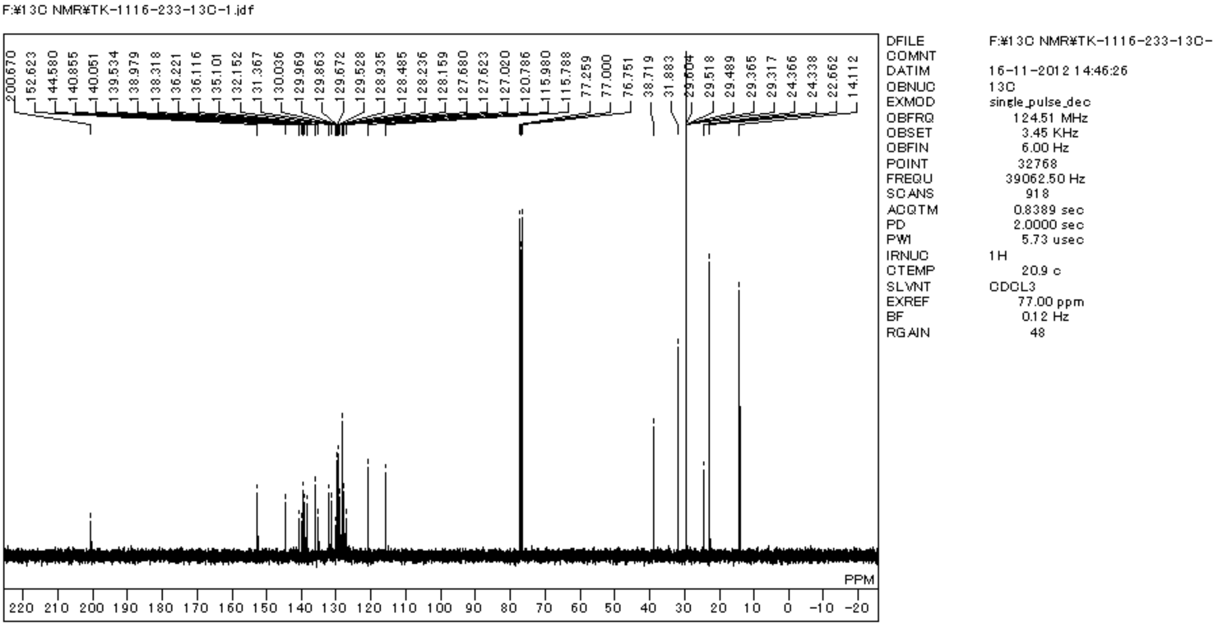
F:¥13C NMR¥TK-1116-233-13C-1.jdf
DFILE F:¥13C NMR¥TK-1116-233-13C-
COMNT
DATIM 16-11-2012 14:46:26
OBNUC 13C
EXMOD single_pulse_dec
OBFRQ 124.51 MHz
OBSET 3.45 KHz
OBFIN 6.00 Hz
POINT 32768
FREQU 39062.50 Hz
SCANS 918
ACQTM 0.8389 sec
PD 2.0000 sec
PW1 5.73 usec
IRNUC 1H
CTEMP 20.9 c
SLVNT CDCL3
EXREF 77.00 ppm
BF 0.12 Hz
RGAIN 48
PPM

6

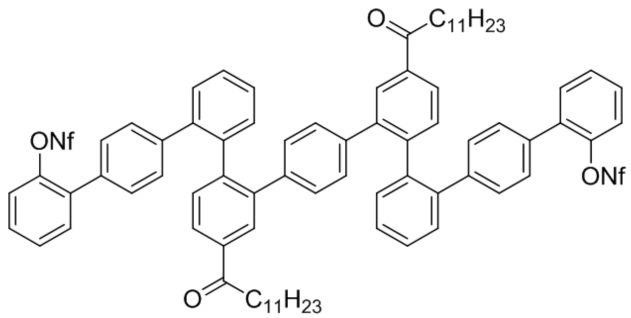
O
C11H23
ONf
ONf
O
C11H23

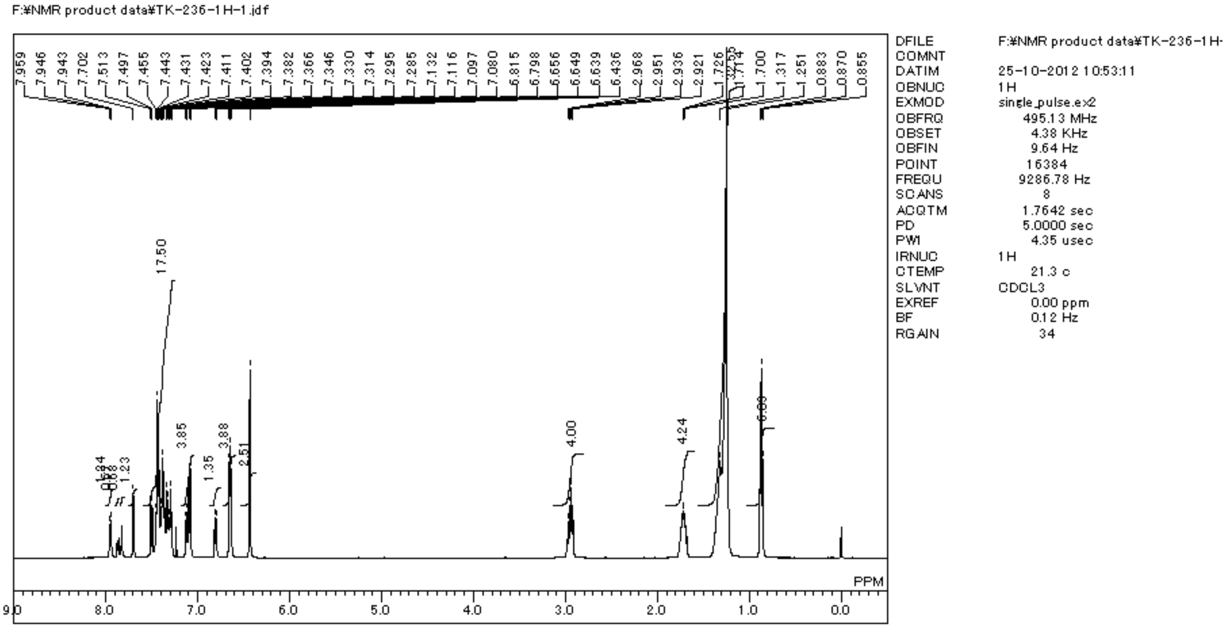
F:¥NMR product data¥TK-236-1H-1.jdf
7.959
7.946
7.943
7.702
7.513
7.497
7.455
7.443
7.431
7.423
7.411
7.402
7.394
7.382
7.366
7.346
7.330
7.314
7.295
7.285
7.132
7.116
7.097
7.080
6.815
6.798
6.656
6.649
6.639
6.436
2.968
2.951
2.936
2.921
1.726
1.714
1.700
1.317
1.251
0.883
0.870
0.855
17.50
3.85
3.88
1.35
2.51
1.23
4.00
4.24
PPM
9.0
8.0
7.0
6.0
5.0
4.0
3.0
2.0
1.0
0.0
DFILE F:¥NMR product data¥TK-236-1H-
COMNT
DATIM 25-10-2012 10:53:11
OBNUC 1H
EXMOD single_pulse.ex2
OBFRQ 495.13 MHz
OBSET 4.38 KHz
OBFIN 9.64 Hz
POINT 16384
FREQU 9286.78 Hz
SCANS 8
ACQTM 1.7642 sec
PD 5.0000 sec
PW1 4.35 usec
IRNUC 1H
CTEMP 21.3 c
SLVNT CDCL3
EXREF 0.00 ppm
BF 0.12 Hz
RGAIN 34

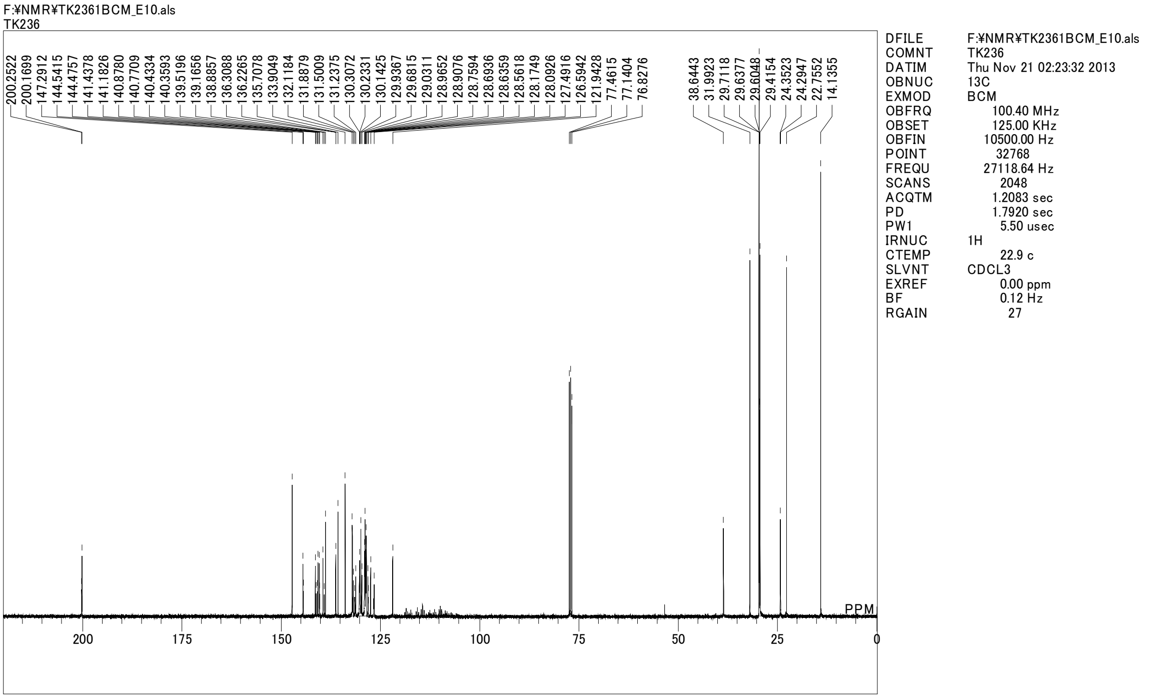
F:¥NMR¥TK2361BCM_E10.als
TK236
200.2522
200.1699
147.2912
144.5415
144.4757
141.4378
141.1826
140.8780
140.7709
140.4334
140.3593
139.5196
139.1656
138.8857
136.3088
136.2265
135.7078
133.9049
132.1184
131.8879
131.5009
131.2375
130.3072
130.2331
130.1425
129.9367
129.6815
129.0311
128.9652
128.9076
128.7594
128.6936
128.6359
128.5618
128.1749
128.0926
127.4916
126.5942
121.9428
77.4615
77.1404
76.8276
38.6443
31.9923
29.7118
29.6377
29.6048
29.4154
24.3523
24.2947
22.7552
14.1355
PPM
200
175
150
125
100
75
50
25
0
DFILE F:¥NMR¥TK2361BCM_E10.als
COMNT TK236
DATIM Thu Nov 21 02:23:32 2013
OBNUC 13C
EXMOD BCM
OBFRQ 100.40 MHz
OBSET 125.00 KHz
OBFIN 10500.00 Hz
POINT 32768
FREQU 27118.64 Hz
SCANS 2048
ACQTM 1.2083 sec
PD 1.7920 sec
PW1 5.50 usec
IRNUC 1H
CTEMP 22.9 c
SLVNT CDCL3
EXREF 0.00 ppm
BF 0.12 Hz
RGAIN 27

7

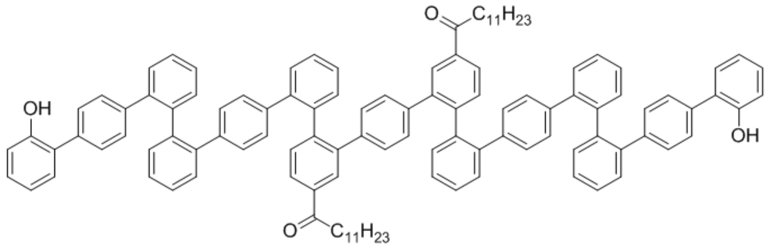
OH
C11H23
OH

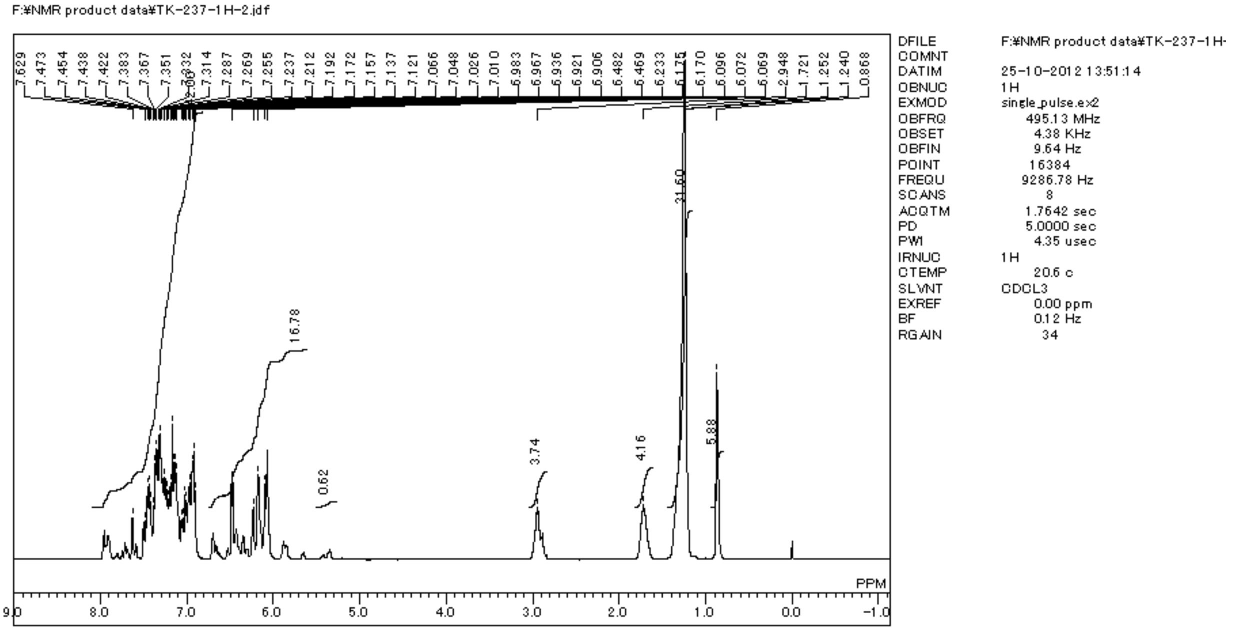
F:¥NMR product data¥TK-237-1H-2.jdf
DFILE F:¥NMR product data¥TK-237-1H-
COMNT
DATIM 25-10-2012 13:51:14
OBNUC 1H
EXMOD single_pulse.ex2
OBFRQ 495.13 MHz
OBSET 4.38 KHz
OBFIN 9.64 Hz
POINT 16384
FREQU 9286.78 Hz
SCANS 8
ACQTM 1.7642 sec
PD 5.0000 sec
PW1 4.35 usec
IRNUC 1H
CTEMP 20.6 c
SLVNT CDCL3
EXREF 0.00 ppm
BF 0.12 Hz
RGAIN 34
PPM

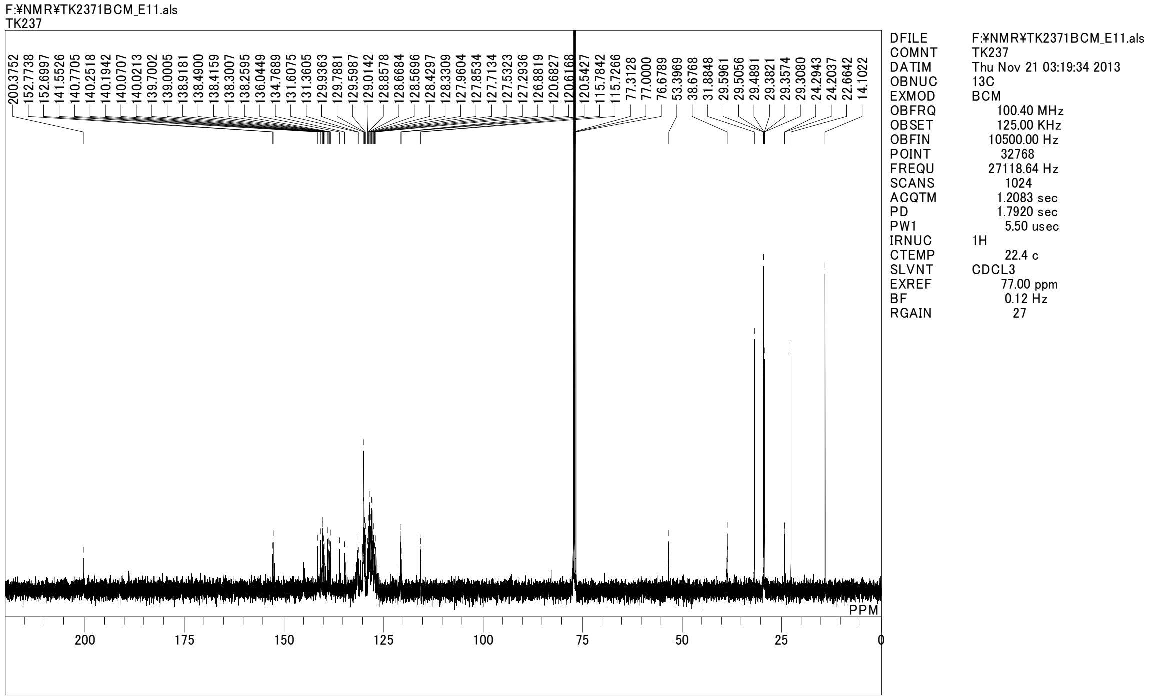
F:¥NMR¥TK2371BCM_E11.als
TK237
DFILE F:¥NMR¥TK2371BCM_E11.als
COMNT TK237
DATIM Thu Nov 21 03:19:34 2013
OBNUC 13C
EXMOD BCM
OBFRQ 100.40 MHz
OBSET 125.00 KHz
OBFIN 10500.00 Hz
POINT 32768
FREQU 27118.64 Hz
SCANS 1024
ACQTM 1.2083 sec
PD 1.7920 sec
PW1 5.50 usec
IRNUC 1H
CTEMP 22.4 c
SLVNT CDCL3
EXREF 77.00 ppm
BF 0.12 Hz
RGAIN 27
PPM

8

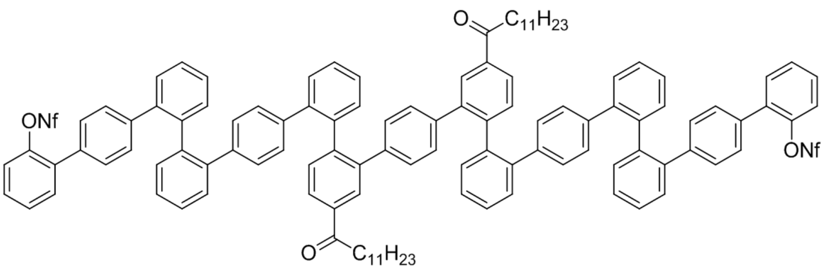
ONf
ONf
$C_{11}H_{23}$
$C_{11}H_{23}$

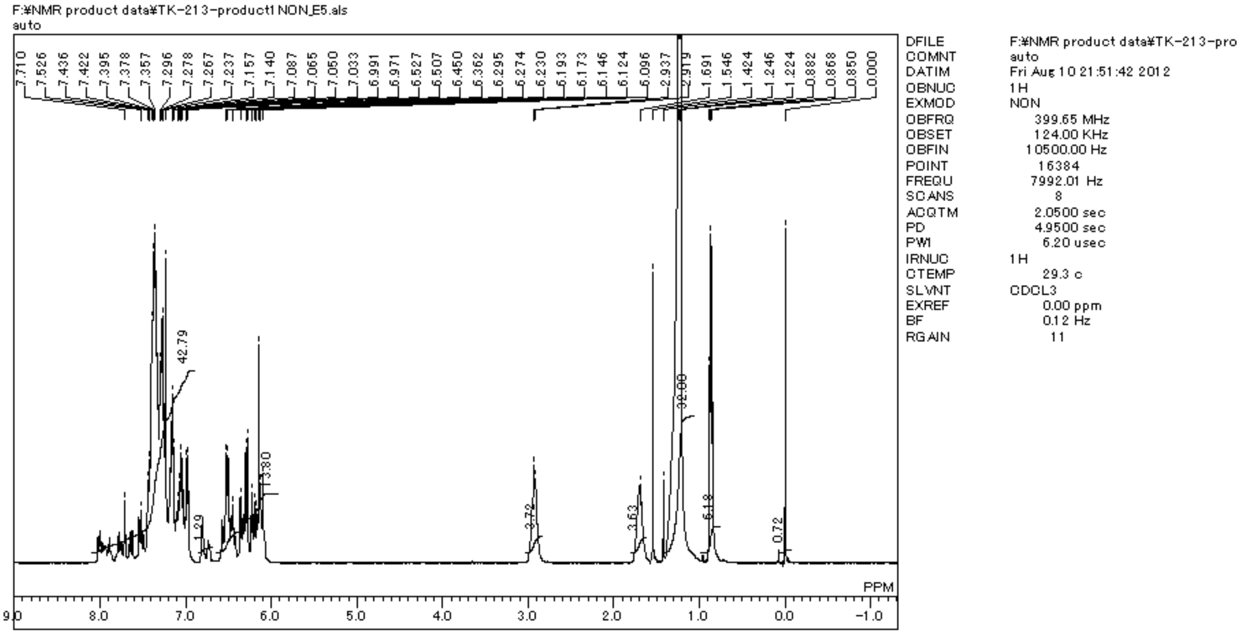
F:¥NMR product data¥TK-213-product1 NON_E5.als
auto
DFILE F:¥NMR product data¥TK-213-pro
COMNT auto
DATIM Fri Aug 10 21:51:42 2012
OBNUC 1H
EXMOD NON
OBFRQ 399.65 MHz
OBSET 124.00 KHz
OBFIN 10500.00 Hz
POINT 16384
FREQU 7992.01 Hz
SCANS 8
ACQTM 2.0500 sec
PD 4.9500 sec
PW1 6.20 usec
IRNUC 1H
CTEMP 29.3 c
SLVNT CDCL3
EXREF 0.00 ppm
BF 0.12 Hz
RGAIN 11
PPM

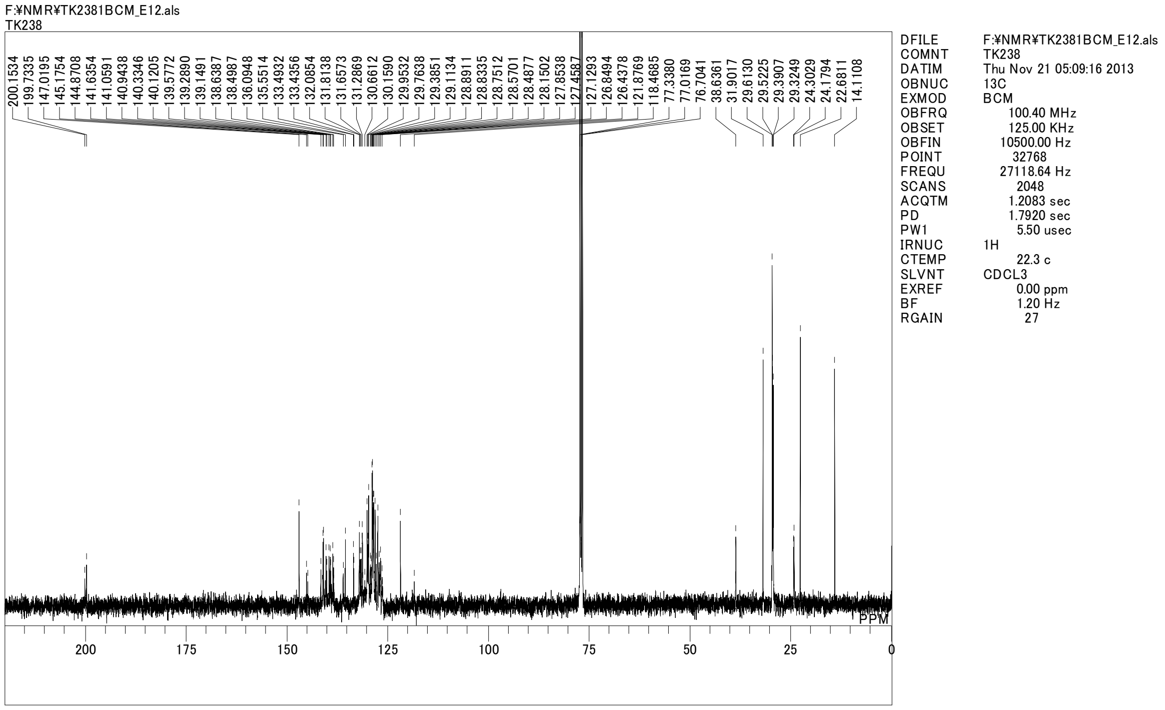
F:¥NMR¥TK2381BCM_E12.als
TK238
DFILE F:¥NMR¥TK2381BCM_E12.als
COMNT TK238
DATIM Thu Nov 21 05:09:16 2013
OBNUC 13C
EXMOD BCM
OBFRQ 100.40 MHz
OBSET 125.00 KHz
OBFIN 10500.00 Hz
POINT 32768
FREQU 27118.64 Hz
SCANS 2048
ACQTM 1.2083 sec
PD 1.7920 sec
PW1 5.50 usec
IRNUC 1H
CTEMP 22.3 c
SLVNT CDCL3
EXREF 0.00 ppm
BF 1.20 Hz
RGAIN 27
PPM

9

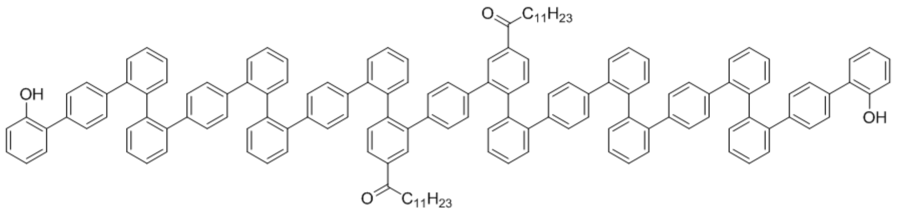
O
C11H23
OH
OH
O
C11H23

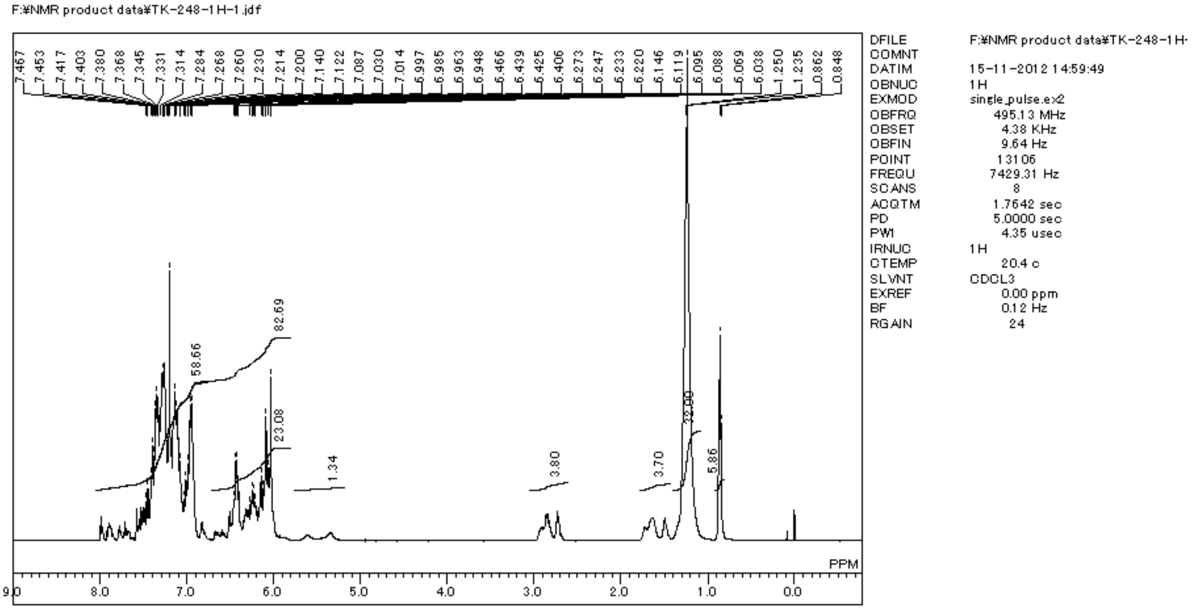
F:¥NMR product data¥TK-248-1H-1.jdf
7.457
7.453
7.417
7.403
7.380
7.368
7.345
7.331
7.314
7.284
7.268
7.260
7.230
7.214
7.200
7.140
7.122
7.087
7.030
7.014
6.997
6.985
6.963
6.948
6.456
6.439
6.425
6.406
6.273
6.247
6.233
6.220
6.146
6.119
6.095
6.088
6.069
6.038
1.250
1.235
0.862
0.848
82.69
58.66
23.08
1.34
3.80
3.70
32.00
5.86
PPM
9.0
8.0
7.0
6.0
5.0
4.0
3.0
2.0
1.0
0.0
DFILE F:¥NMR product data¥TK-248-1H-
COMNT
DATIM 15-11-2012 14:59:49
OBNUC 1H
EXMOD single_pulse.ex2
OBFRQ 495.13 MHz
OBSET 4.38 KHz
OBFIN 9.64 Hz
POINT 13106
FREQU 7429.31 Hz
SCANS 8
ACQTM 1.7642 sec
PD 5.0000 sec
PW1 4.35 usec
IRNUC 1H
CTEMP 20.4 c
SLVNT CDCL3
EXREF 0.00 ppm
BF 0.12 Hz
RGAIN 24

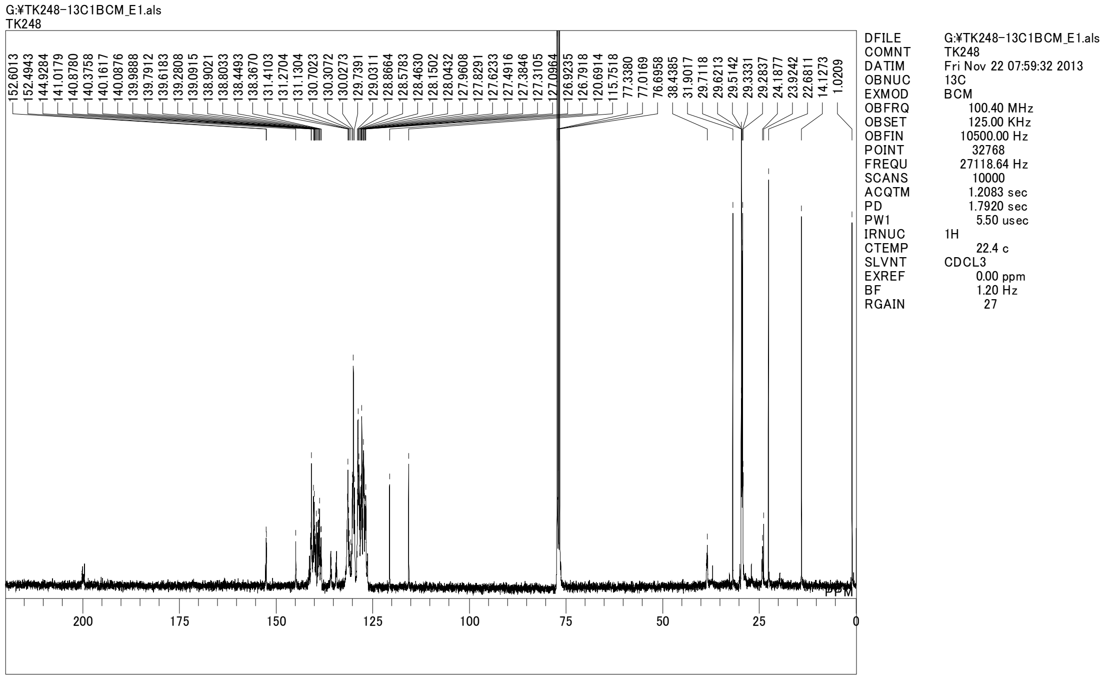
G:¥TK248-13C1BCM_E1.als
TK248
152.6013
152.4943
144.9284
141.0179
140.8780
140.3758
140.1617
140.0876
139.9888
139.7912
139.6183
139.2808
139.0915
138.9021
138.8033
138.4493
138.3670
131.4103
131.2704
131.1304
130.7023
130.3072
130.0273
129.7391
129.0311
128.8664
128.5783
128.4630
128.1502
128.0432
127.9608
127.8291
127.6233
127.4916
127.3846
127.3105
127.0964
126.9235
126.7918
120.6914
115.7518
77.3380
77.0169
76.6958
38.4385
31.9017
29.7118
29.6213
29.5142
29.3331
29.2837
24.1877
23.9242
22.6811
14.1273
1.0209
PPM
200
175
150
125
100
75
50
25
0
DFILE G:¥TK248-13C1BCM_E1.als
COMNT TK248
DATIM Fri Nov 22 07:59:32 2013
OBNUC 13C
EXMOD BCM
OBFRQ 100.40 MHz
OBSET 125.00 KHz
OBFIN 10500.00 Hz
POINT 32768
FREQU 27118.64 Hz
SCANS 10000
ACQTM 1.2083 sec
PD 1.7920 sec
PW1 5.50 usec
IRNUC 1H
CTEMP 22.4 c
SLVNT CDCL3
EXREF 0.00 ppm
BF 1.20 Hz
RGAIN 27

10

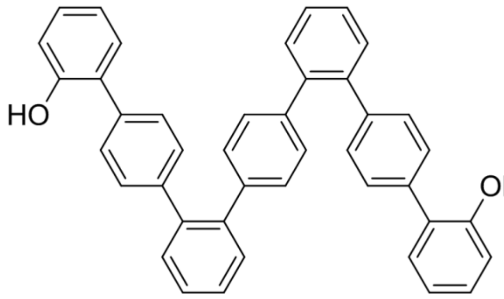
HO
OH

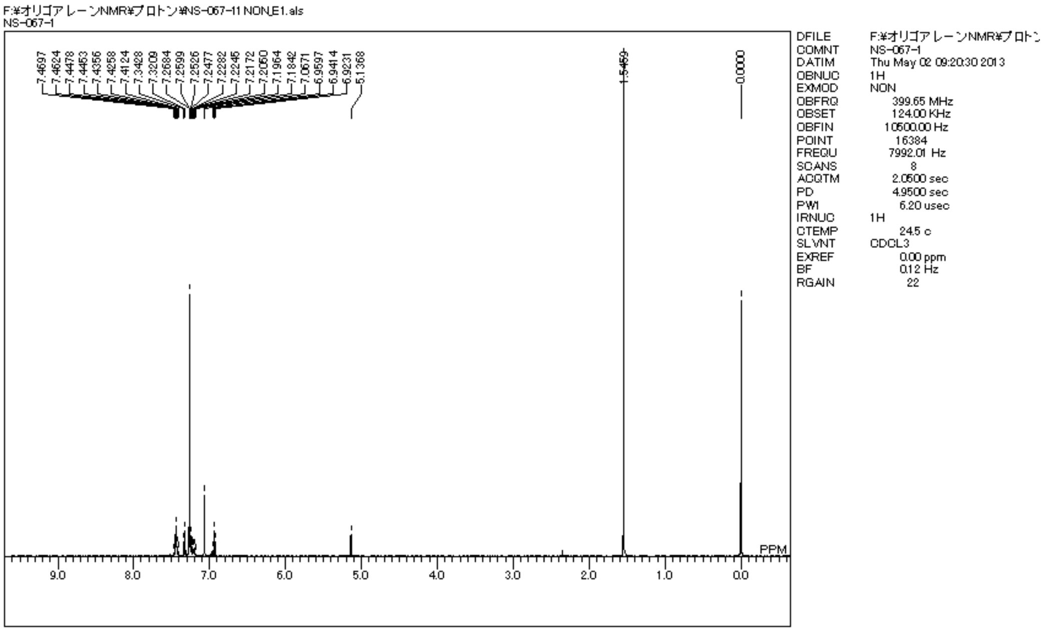
F:¥オリゴアレーンNMR¥プロトン¥NS-067-11 NON_E1.als
NS-067-1
7.4697
7.4624
7.4478
7.4453
7.4356
7.4258
7.4124
7.3428
7.3209
7.2684
7.2599
7.2526
7.2477
7.2282
7.2245
7.2172
7.2050
7.1964
7.1842
7.0671
6.9597
6.9414
6.9231
5.1368
1.5459
0.0000
PPM
9.0
8.0
7.0
6.0
5.0
4.0
3.0
2.0
1.0
0.0
DFILE F:¥オリゴアレーンNMR¥プロトン
COMNT NS-067-1
DATIM Thu May 02 09:20:30 2013
OBNUC 1H
EXMOD NON
OBFRQ 399.65 MHz
OBSET 124.00 KHz
OBFIN 10500.00 Hz
POINT 16384
FREQU 7992.01 Hz
SCANS 8
ACQTM 2.0500 sec
PD 4.9500 sec
PW1 6.20 usec
IRNUC 1H
CTEMP 24.5 c
SLVNT CDCL3
EXREF 0.00 ppm
BF 0.12 Hz
RGAIN 22

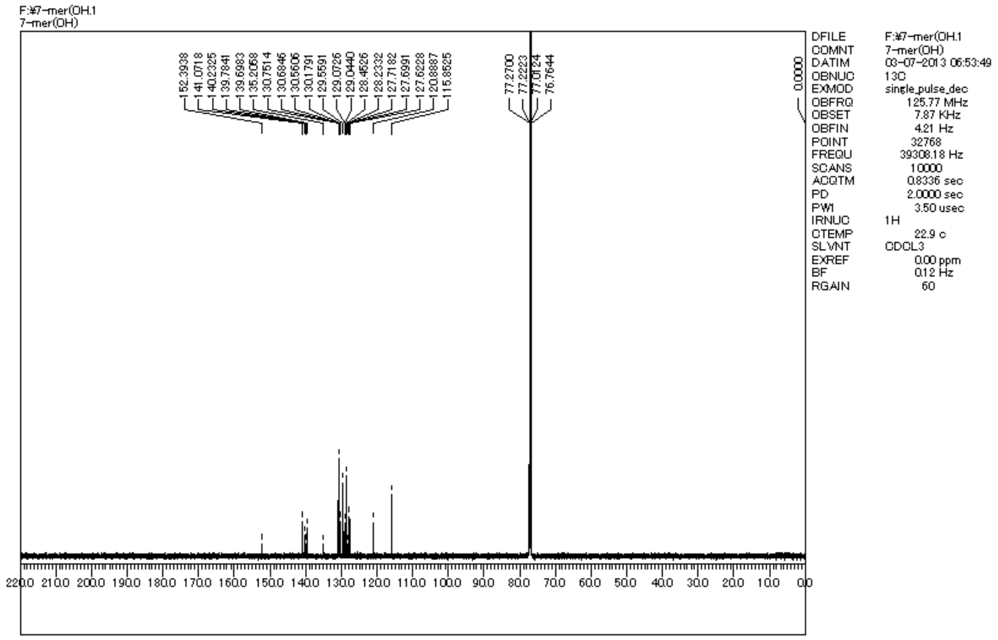
F:¥7-mer(OH.1
7-mer(OH)
152.3938
141.0718
140.2325
139.7841
139.6983
135.2068
130.7514
130.6846
130.5606
130.1791
129.5591
129.0726
129.0440
128.4626
128.2332
127.7182
127.6991
127.6228
120.8887
115.8525
77.2700
77.2223
77.0124
76.7644
0.0000
220.0 210.0 200.0 190.0 180.0 170.0 160.0 150.0 140.0 130.0 120.0 110.0 100.0 90.0 80.0 70.0 60.0 50.0 40.0 30.0 20.0 10.0 0.0
DFILE F:¥7-mer(OH.1
COMNT 7-mer(OH)
DATIM 03-07-2013 06:53:49
OBNUC 13C
EXMOD single_pulse_dec
OBFRQ 125.77 MHz
OBSET 7.87 KHz
OBFIN 4.21 Hz
POINT 32768
FREQU 39308.18 Hz
SCANS 10000
ACQTM 0.8336 sec
PD 2.0000 sec
PW1 3.50 usec
IRNUC 1H
CTEMP 22.9 c
SLVNT CDCL3
EXREF 0.00 ppm
BF 0.12 Hz
RGAIN 60

11

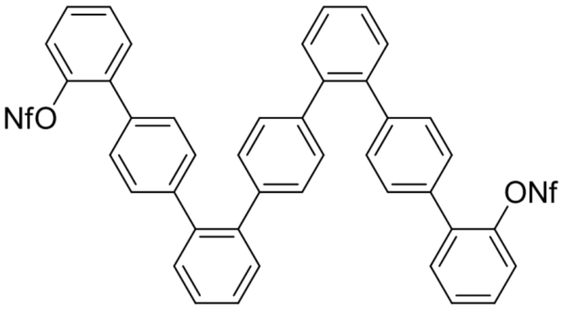
NfO
ONf

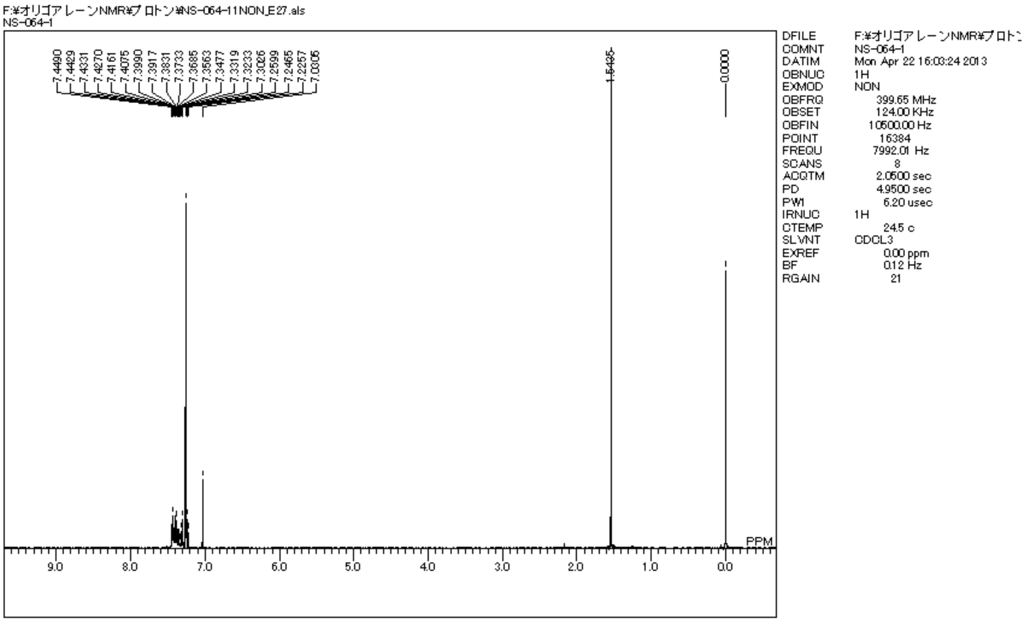
F:¥オリゴアレーンNMR¥プロトン¥NS-064-11NON_E27.als
NS-064-1
7.4490
7.4429
7.4331
7.4270
7.4161
7.4075
7.3990
7.3917
7.3831
7.3733
7.3685
7.3563
7.3477
7.3319
7.3233
7.3026
7.2599
7.2465
7.2257
7.0306
1.5435
0.0000
PPM
9.0 8.0 7.0 6.0 5.0 4.0 3.0 2.0 1.0 0.0
DFILE F:¥オリゴアレーンNMR¥プロト
COMNT NS-064-1
DATIM Mon Apr 22 16:03:24 2013
OBNUC 1H
EXMOD NON
OBFRQ 399.65 MHz
OBSET 124.00 KHz
OBFIN 10500.00 Hz
POINT 16384
FREQU 7992.01 Hz
SCANS 8
ACQTM 2.0500 sec
PD 4.9500 sec
PW1 6.20 usec
IRNUC 1H
CTEMP 24.5 c
SLVNT CDCL3
EXREF 0.00 ppm
BF 0.12 Hz
RGAIN 21

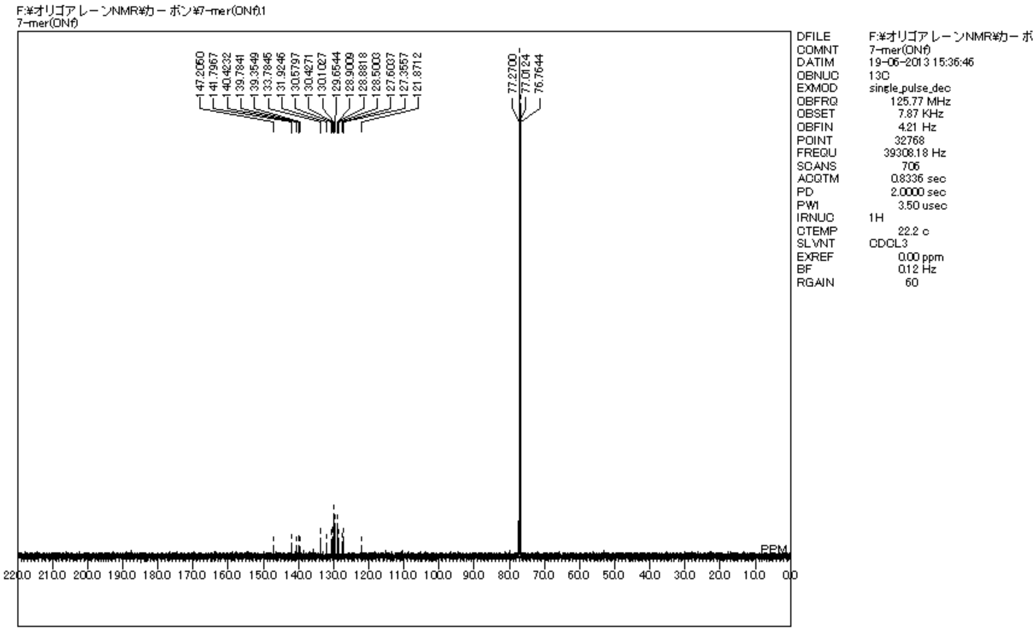
F:¥オリゴアレーンNMR¥カーボン¥7-mer(ONf).1
7-mer(ONf)
147.2050
141.7967
140.4232
139.7841
139.3549
133.7846
131.9246
130.5797
130.4271
130.1027
129.6544
128.9009
128.8818
128.5003
127.6037
127.3557
121.8712
77.2700
77.0124
76.7644
PPM
220.0 210.0 200.0 190.0 180.0 170.0 160.0 150.0 140.0 130.0 120.0 110.0 100.0 90.0 80.0 70.0 60.0 50.0 40.0 30.0 20.0 10.0 0.0
DFILE F:¥オリゴアレーンNMR¥カーボ
COMNT 7-mer(ONf)
DATIM 19-06-2013 15:36:46
OBNUC 13C
EXMOD single_pulse_dec
OBFRQ 125.77 MHz
OBSET 7.87 KHz
OBFIN 4.21 Hz
POINT 32768
FREQU 39308.18 Hz
SCANS 706
ACQTM 0.8336 sec
PD 2.0000 sec
PW1 3.50 usec
IRNUC 1H
CTEMP 22.2 c
SLVNT CDCL3
EXREF 0.00 ppm
BF 0.12 Hz
RGAIN 60

12

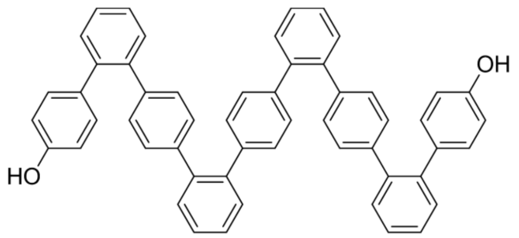
OH
HO

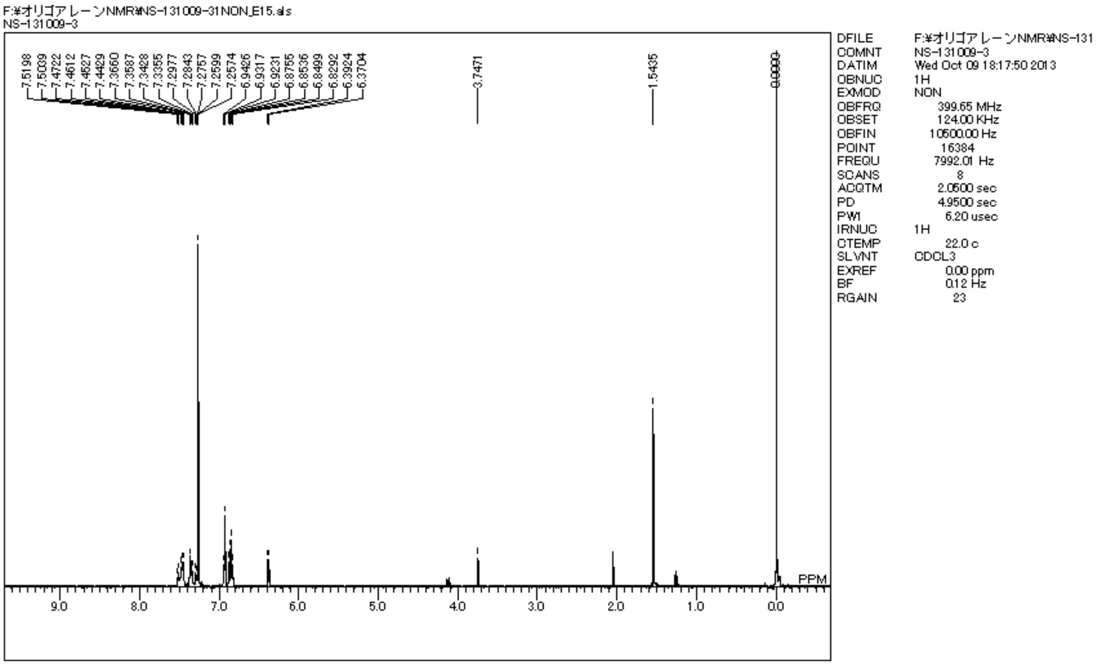
F:¥オリゴアレーンNMR¥NS-131009-31NON_E15.als
NS-131009-3
7.5198
7.5039
7.4722
7.4612
7.4527
7.4429
7.3660
7.3587
7.3428
7.3355
7.2977
7.2843
7.2757
7.2599
7.2574
6.9426
6.9317
6.9231
6.8755
6.8536
6.8499
6.8292
6.3924
6.3704
3.7471
1.5435
0.0000
PPM
9.0 8.0 7.0 6.0 5.0 4.0 3.0 2.0 1.0 0.0
DFILE F:¥オリゴアレーンNMR¥NS-131
COMNT NS-131009-3
DATIM Wed Oct 09 18:17:50 2013
OBNUC 1H
EXMOD NON
OBFRQ 399.65 MHz
OBSET 124.00 KHz
OBFIN 10500.00 Hz
POINT 16384
FREQU 7992.01 Hz
SCANS 8
ACQTM 2.0500 sec
PD 4.9500 sec
PW1 6.20 usec
IRNUC 1H
CTEMP 22.0 c
SLVNT CDCL3
EXREF 0.00 ppm
BF 0.12 Hz
RGAIN 23

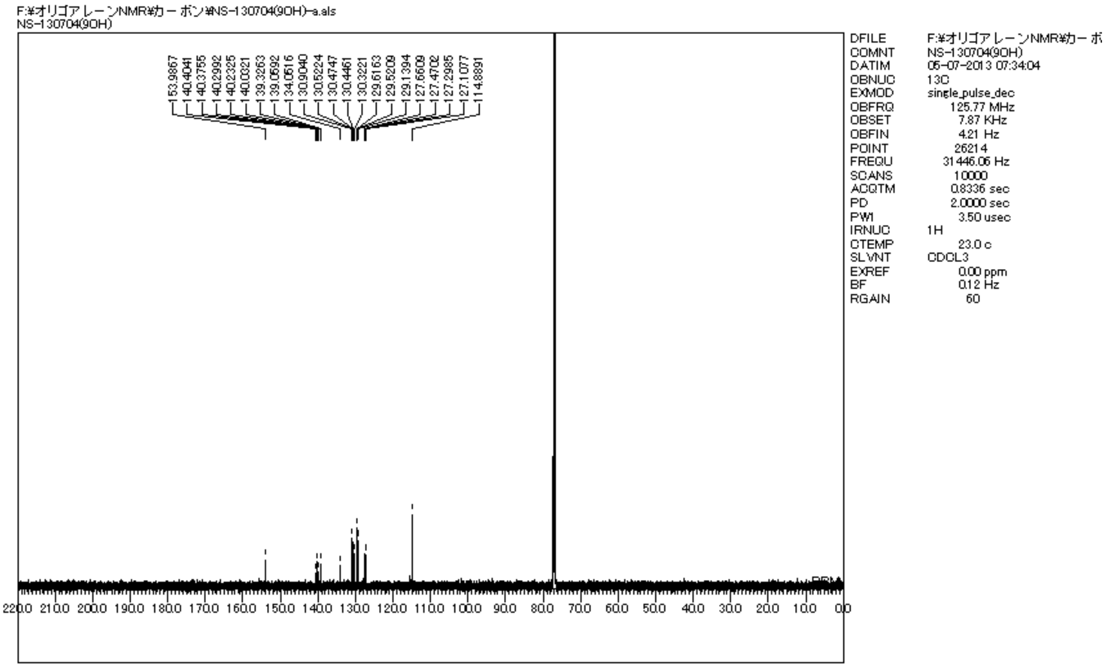
F:¥オリゴアレーンNMR¥カーボン¥NS-130704(9OH)-a.als
NS-130704(9OH)
153.9867
140.4041
140.3755
140.2992
140.2325
140.0321
139.3263
139.0592
134.0516
130.9040
130.5224
130.4747
130.4461
130.3221
129.6163
129.5209
129.1394
127.6609
127.4702
127.2985
127.1077
114.8891
PPM
220.0 210.0 200.0 190.0 180.0 170.0 160.0 150.0 140.0 130.0 120.0 110.0 100.0 90.0 80.0 70.0 60.0 50.0 40.0 30.0 20.0 10.0 0.0
DFILE F:¥オリゴアレーンNMR¥カーボ
COMNT NS-130704(9OH)
DATIM 05-07-2013 07:34:04
OBNUC 13C
EXMOD single_pulse_dec
OBFRQ 125.77 MHz
OBSET 7.87 KHz
OBFIN 4.21 Hz
POINT 26214
FREQU 31446.06 Hz
SCANS 10000
ACQTM 0.8336 sec
PD 2.0000 sec
PW1 3.50 usec
IRNUC 1H
CTEMP 23.0 c
SLVNT CDCL3
EXREF 0.00 ppm
BF 0.12 Hz
RGAIN 60

13

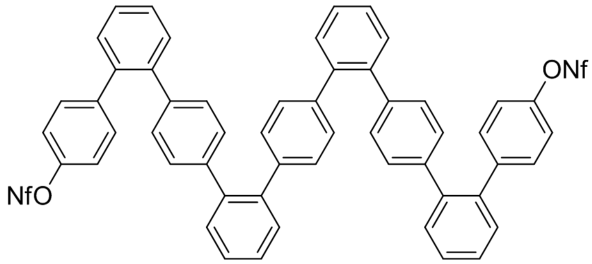
ONf
NfO

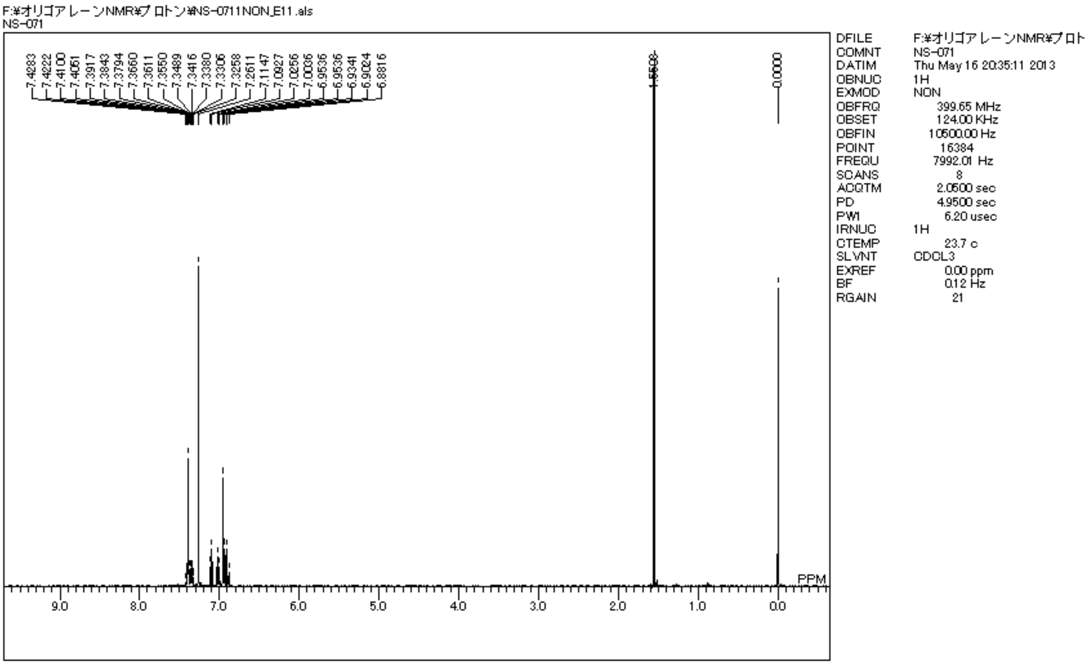
F:¥オリゴアレーンNMR¥プロトン¥NS-0711NON_E11.als
NS-071
7.4283
7.4222
7.4100
7.4061
7.3917
7.3843
7.3794
7.3660
7.3611
7.3550
7.3489
7.3416
7.3380
7.3306
7.3258
7.2611
7.1147
7.0927
7.0256
7.0036
6.9536
6.9341
6.9024
6.8816
1.5508
0.0000
PPM
9.0 8.0 7.0 6.0 5.0 4.0 3.0 2.0 1.0 0.0
DFILE F:¥オリゴアレーンNMR¥プロト
COMNT NS-071
DATIM Thu May 16 20:35:11 2013
OBNUC 1H
EXMOD NON
OBFRQ 399.65 MHz
OBSET 124.00 KHz
OBFIN 10500.00 Hz
POINT 16384
FREQU 7992.01 Hz
SCANS 8
ACQTM 2.0500 sec
PD 4.9500 sec
PW1 6.20 usec
IRNUC 1H
CTEMP 23.7 c
SLVNT CDCL3
EXREF 0.00 ppm
BF 0.12 Hz
RGAIN 21

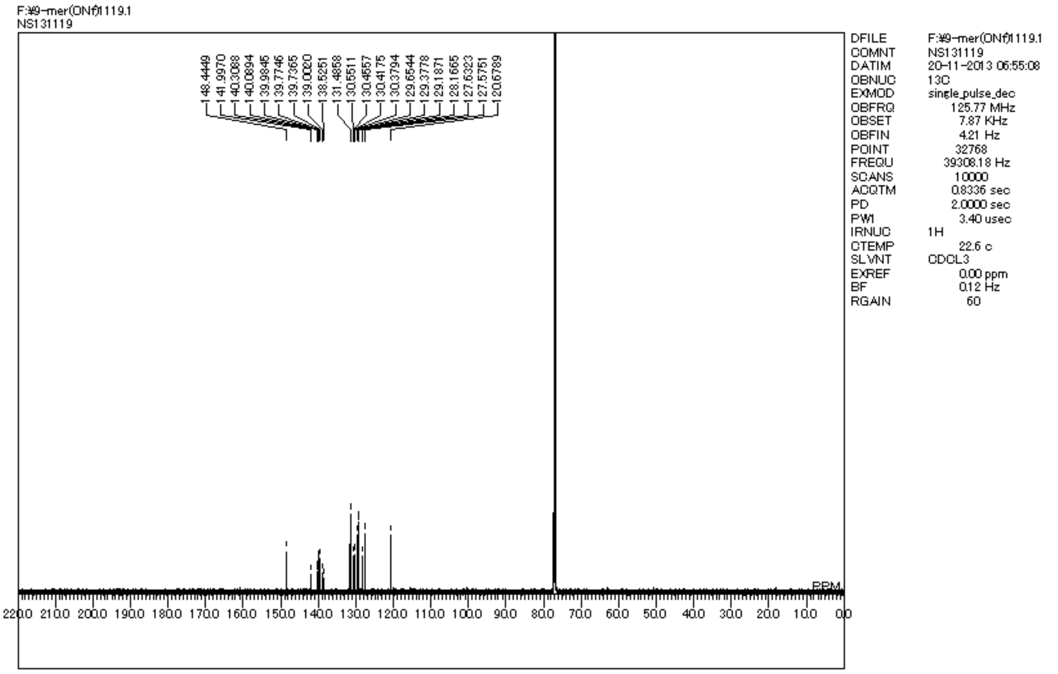
F:¥9-mer(ONf)1119.1
NS131119
148.4449
141.9970
140.3088
140.0894
139.9845
139.7746
139.7365
139.0020
138.5251
131.4858
130.5511
130.4557
130.4175
130.3794
129.6544
129.3778
129.1871
128.1665
127.6323
127.5751
120.6789
PPM
220.0 210.0 200.0 190.0 180.0 170.0 160.0 150.0 140.0 130.0 120.0 110.0 100.0 90.0 80.0 70.0 60.0 50.0 40.0 30.0 20.0 10.0 0.0
DFILE F:¥9-mer(ONf)1119.1
COMNT NS131119
DATIM 20-11-2013 06:55:08
OBNUC 13C
EXMOD single_pulse_dec
OBFRQ 125.77 MHz
OBSET 7.87 KHz
OBFIN 4.21 Hz
POINT 32768
FREQU 39308.18 Hz
SCANS 10000
ACQTM 0.8336 sec
PD 2.0000 sec
PW1 3.40 usec
IRNUC 1H
CTEMP 22.6 c
SLVNT CDCL3
EXREF 0.00 ppm
BF 0.12 Hz
RGAIN 60

14

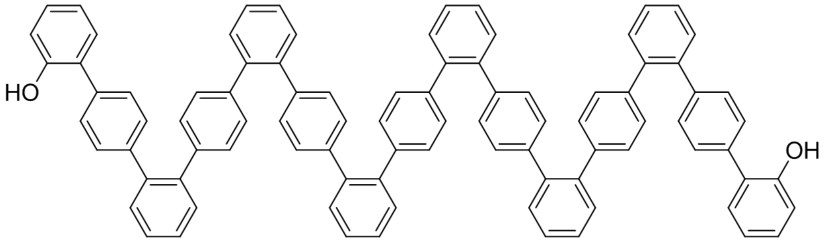
HO
OH

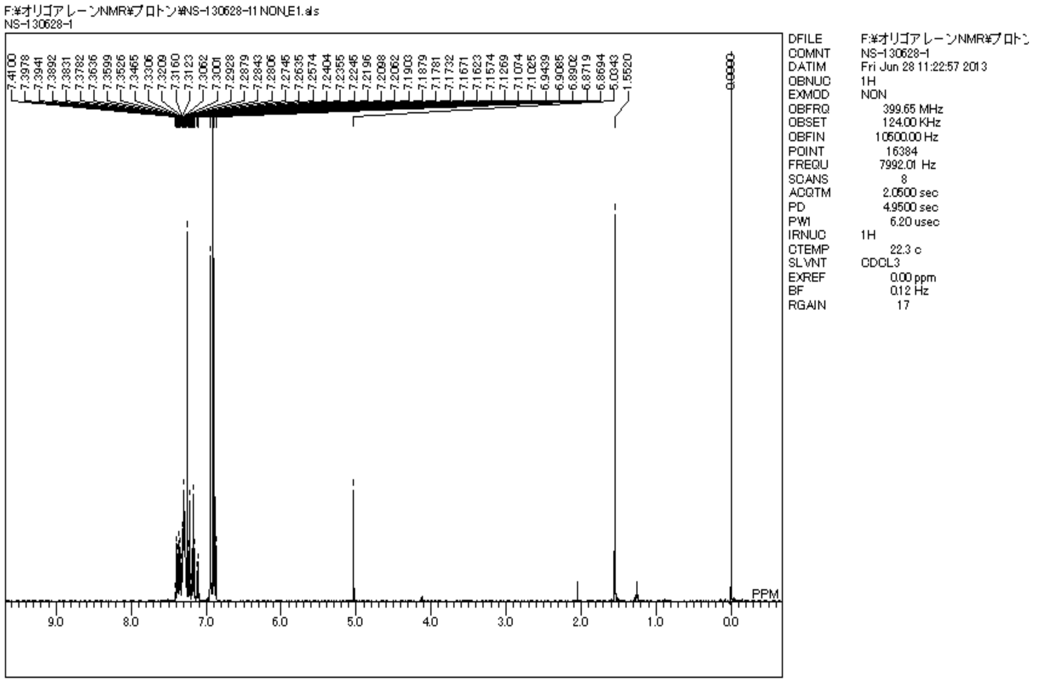
NS-130628-1
DFILE
COMNT NS-130628-1
DATIM Fri Jun 28 11:22:57 2013
OBNUC 1H
EXMOD NON
OBFRQ 399.65 MHz
OBSET 124.00 KHz
OBFIN 10500.00 Hz
POINT 16384
FREQU 7992.01 Hz
SCANS 8
ACQTM 2.0500 sec
PD 4.9500 sec
PW1 6.20 usec
IRNUC 1H
CTEMP 22.3 c
SLVNT CDCL3
EXREF 0.00 ppm
BF 0.12 Hz
RGAIN 17
PPM

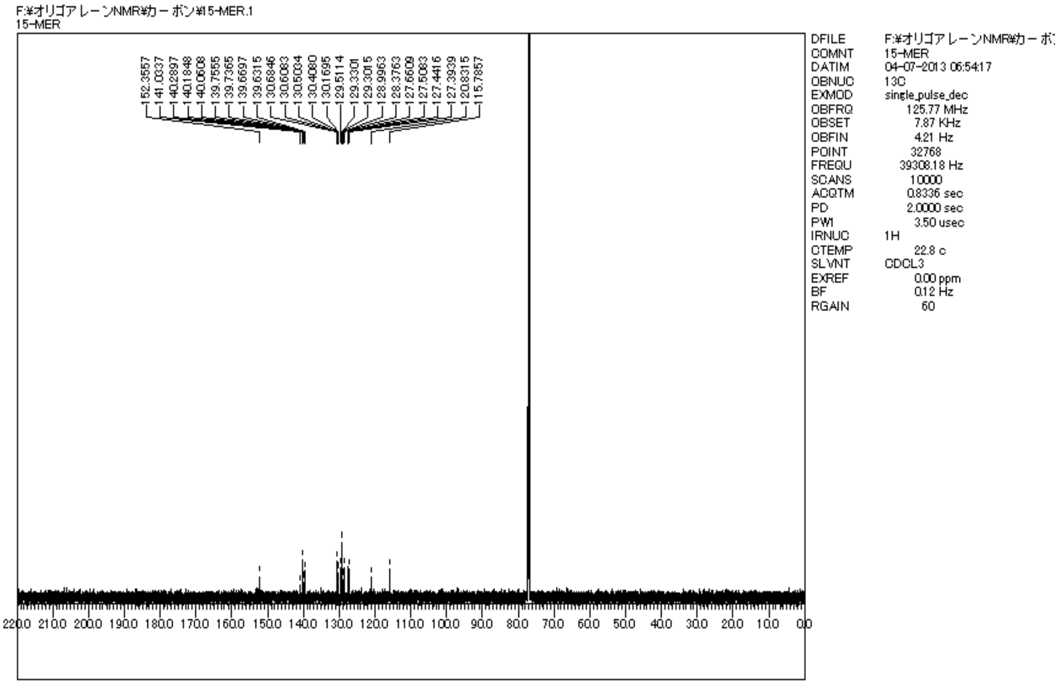
15-MER
DFILE
COMNT 15-MER
DATIM 04-07-2013 06:54:17
OBNUC 13C
EXMOD single_pulse_dec
OBFRQ 125.77 MHz
OBSET 7.87 KHz
OBFIN 4.21 Hz
POINT 32768
FREQU 39308.18 Hz
SCANS 10000
ACQTM 0.8336 sec
PD 2.0000 sec
PW1 3.50 usec
IRNUC 1H
CTEMP 22.8 c
SLVNT CDCL3
EXREF 0.00 ppm
BF 0.12 Hz
RGAIN 60
